# Research on the Mechanism of Natural Products Acting on Chronic Obstructive Pulmonary Disease

**DOI:** 10.1002/fsn3.71965

**Published:** 2026-07-02

**Authors:** Shuna Wei, Xiaoju Liu

**Affiliations:** ^1^ The First Clinical Medical College Lanzhou University Lanzhou Gansu China; ^2^ Department of Gerontal Respiratory Medicine The First Hospital of Lanzhou University Lanzhou Gansu China

**Keywords:** antioxidants, chronic obstructive pulmonary disease, multi‐targets, natural products, therapeutic mechanisms

## Abstract

Chronic obstructive pulmonary disease (COPD) is one of the major health problems worldwide. Its pathological process involves multiple mechanisms such as oxidative stress, chronic airway inflammation, airway remodeling, and excessive mucus secretion. Current clinical therapeutic drugs have limitations in curbing the progression of the disease. An increasing number of studies have shown that natural products, due to their multi‐target synergistic regulatory characteristics and good biological safety, have shown important research value and application potential in the prevention and treatment of COPD. This article discusses the potential therapeutic effects of about 80 natural compounds from plants, microorganisms, animals, and marine organisms on COPD, and summarizes more than 350 references to analyze their potential mechanisms.

## Graphical abstract

This figure illustrates that approximately 80 natural compounds exert therapeutic effects against chronic obstructive pulmonary disease by targeting pathological mechanisms such as airway inflammation, oxidative stress, airway remodeling, mucus hypersecretion, emphysema, protease–antiprotease imbalance, apoptosis, and autophagy. (Created in https://biorender.com).

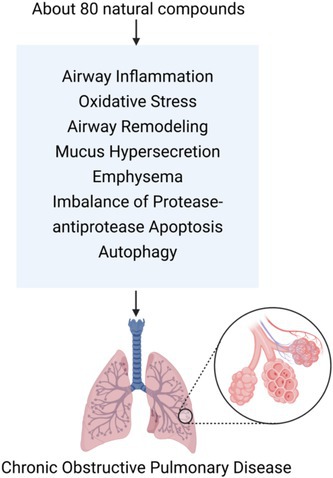

**Abbreviations**
AMPK: AMP‐activated protein kinase
ARE: Antioxidant response element
CHOP: C/EBP homologous protein
FoxO3a: Forkhead box O3a
GPx: Glutathione peroxidase
HIF‐1α: Hypoxia‐inducible factor‐1α
IL‐1β: Interleukin‐1 beta
IL‐6: Interleukin‐6
KC: Keratinocyte‐derived chemokine
NLRP3: Nucleotide‐binding oligomerization domain, leucine‐rich repeat and pyrin domain‐containing 3
Nrf2: Nuclear factor erythroid 2‐related factor 2
PPAR‐γ: Peroxisome proliferator‐activated receptor gamma
RUNX1: Runt‐related transcription factor 1
TIMP‐1: Tissue inhibitor of metalloproteinase 1
TNF‐α: Tumor necrosis factor alpha
TRPV1: Transient receptor potential vanilloid 1
ZO‐1: Zonula occludens‐1

## 1 Introduction

Chronic obstructive pulmonary disease (COPD) is a respiratory disease characterized by persistent airflow limitation, with high prevalence and mortality rates. It has become a major global public health challenge. Epidemiological data show that the global prevalence of COPD is 10.3%, and it continues to increase with the rise in smoking rates and population aging in low‐ and middle‐income countries (Adeloye et al. 2015, 2022; Fallahzadeh et al. 2022). COPD is the third leading cause of death worldwide (World Health Organization 2025), with approximately 3.23 million deaths in 2019, and it is projected to exceed 5.4 million by 2060 (Mathers). The high concealment of early COPD further intensifies the difficulty in disease prevention and control, becoming an important underlying factor for the persistently high mortality rate of COPD (Lei et al. 2024). In terms of economic burden, COPD accounts for 56% of the EU's respiratory disease medical budget each year (about 38.6 billion euros) (World Health Organization 2025). The total cost related to this disease in the United States is expected to reach 800.9 billion US dollars in the next 20 years (Zafari et al. 2021), and it is projected to become the seventh leading disease burden by 2030 (Liao et al. 2024). This highlights the urgency of strengthening its prevention and control.

The onset of COPD is primarily linked to long‐term exposure to toxic particles such as cigarette smoke, occupational dust, and fuel smoke, along with respiratory tract infections (Gayle et al. 2021). These factors can trigger chronic inflammation, leading to airway abnormalities (chronic bronchitis, bronchiolitis) and alveolar destruction (Singh et al. 2025). Pathological changes occur at multiple sites: in the central airways, there is epithelial cell damage, inflammatory cell infiltration, goblet cell hyperplasia, and increased mucus secretion; in the peripheral small airways, lumen constriction occurs; and in the lung parenchyma, typical features include decreased elasticity of alveolar walls and dilation of alveolar sacs (Hogg and Timens 2009; Kim et al. 2024). This pathological process involves several mechanisms including chronic inflammation, oxidative stress, protease‐antiprotease imbalance, fibrosis, and excessive mucus secretion (Zhang, Luo, et al. 2020).

Current COPD treatment combines drug and non‐drug interventions. However, drug therapy has limitations: many patients show low sensitivity to corticosteroids, and inhaled corticosteroids (ICS) are less effective in patients with COPD than in those with asthma (Barnes 2013); while phosphodiesterase 4 (PDE4) inhibitors are restricted in clinical application due to their low therapeutic index and side effects (Vogelmeier et al. 2020). Currently available drug interventions can only provide symptom relief but are limited in their ability to fully control or reverse disease progression (Liao et al. 2024). Additionally, COPD patients often have systemic manifestations such as decreased exercise tolerance, fatigue, osteoporosis, depression, and anxiety in addition to pulmonary symptoms, which seriously affect their quality of life (Agustí et al. 2022; Aldhahir et al. 2020).

**FIGURE 1:**
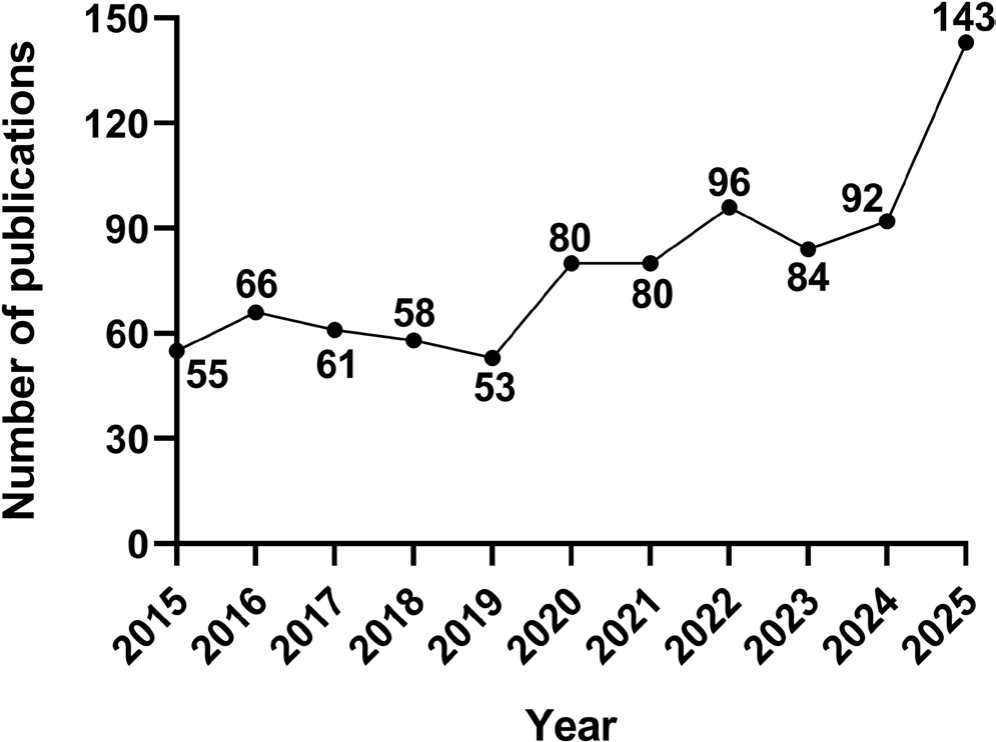
Publication trend of natural products research related to COPD from 2015 to 2025 (created with GraphPad Prism 9.0).

**FIGURE 2:**
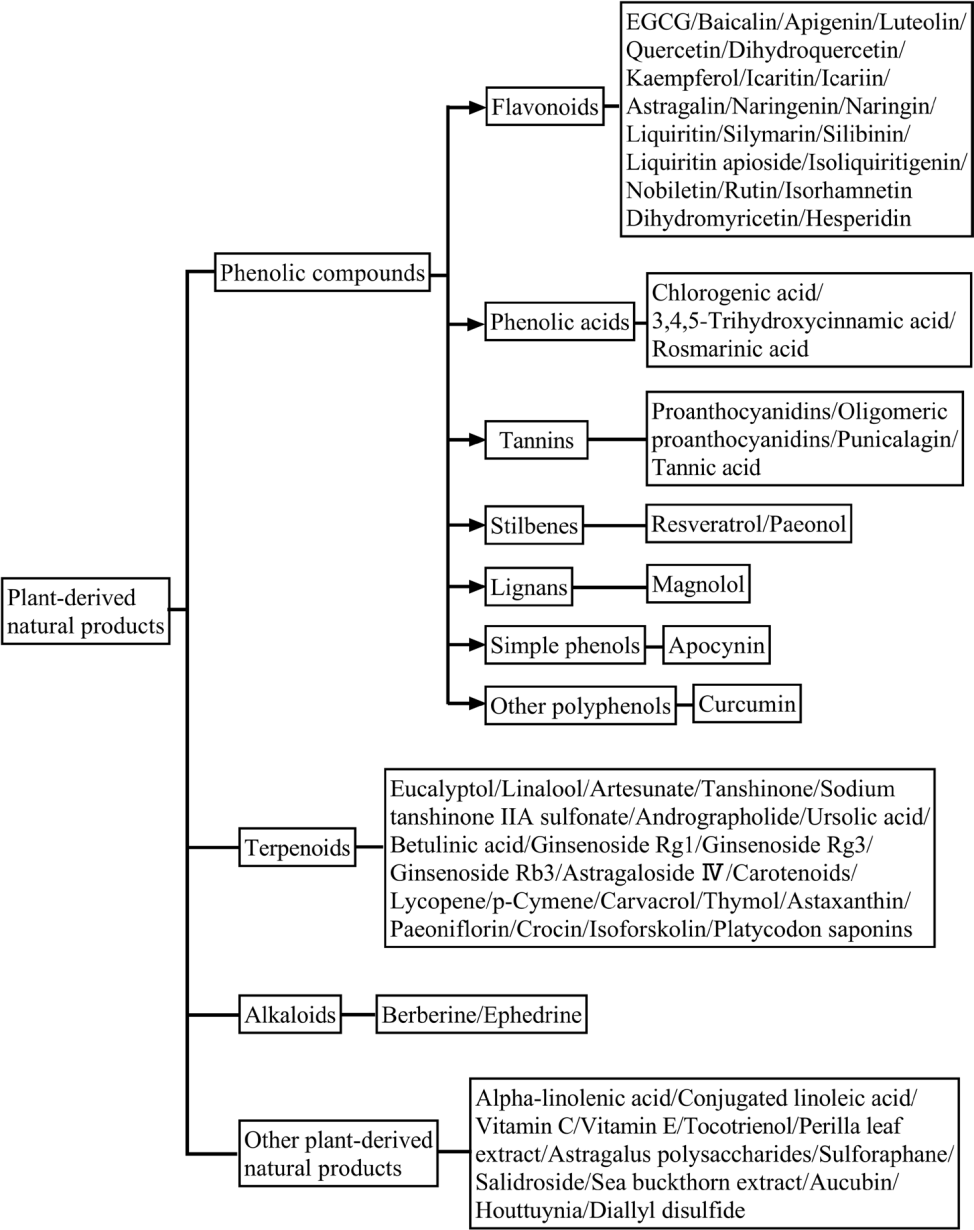
Natural substances from plant sources.

**FIGURE 3:**
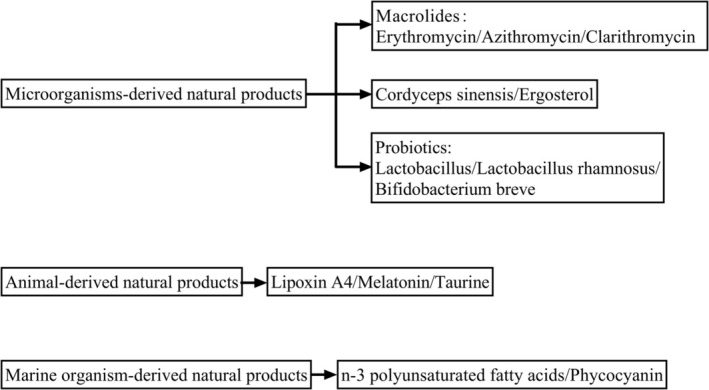
Natural substances from microorganisms, animals, and marine organisms sources.

In recent years, the in‐depth investigation of the pathological mechanisms underlying COPD has garnered significant attention regarding the application of natural products (NPs) for its treatment and prevention (Figure 1). NPs are chemical compounds produced by various organisms, including plants, animals, marine, and microorganisms (Sorokina and Steinbeck 2020). Both in vitro and in vivo studies have shown that many NPs have anti‐inflammatory, antioxidant, and lung‐protective effects and can be used in the treatment of COPD. Given the current absence of a comprehensive overview summarizing the interventions provided by NPs from diverse sources on COPD, this review comprehensively summarizes the therapeutic effects of NPs derived from plants (phenolic compounds, terpenoids, alkaloids, polyunsaturated fatty acids, and vitamins), microorganisms (macrolides, Cordyceps sinensis, and probiotics), animals (lipoxin A4, melatonin, and taurine), and marine organisms (n‐3 polyunsaturated fatty acids and phycocyanin) against COPD (Figures 2 and 3; Tables 1, 2, 3, 4, 5, 6). We systematically analyze their pulmonary protective effects mediated through multi‐target mechanisms, including anti‐inflammatory, antioxidant, anti‐fibrotic, and anti‐aging pathways, and discuss recent advances in clinical trials. By integrating data from over 80 natural compounds and more than 350 references, this review aims to provide a theoretical foundation and identify research directions for the development of natural therapeutics in COPD management.

**TABLE 1:** Effects of flavonoids on animal models of COPD.

Subclassification	Substance	Molecular formula	Author(s)	Animal model	Species	Dose	Effect	Mechanism
Flavan‐3‐ols	Epigallocatechin‐3‐gallate	C_22_H_18_O_11_	Liang et al. 2017	CS‐induced COPD model	Sprague–Dawley rats	During the 56‐day CS exposure period, EGCG (50 mg/kg) was administered via oral gavage every other day.	Anti‐inflammatory; anti‐mucus secretion	↑: SOD, CAT; ↓: GST, MCP‐1, neutrophil count, MUC5AC, p‐EGFR, EGFR protein
			Cheng et al. 2025	CS‐ and LPS‐induced AECOPD model	C57BL/6J mice	QKPC was administered via intragastric administration at doses of 5 or 10 g/kg once daily for 5 consecutive days.	Anti‐inflammatory; antioxidant; anti‐mucus secretion	↓: IL‐6, CXCL1, TNF‐α, neutrophil count, MUC5AC, ROS, NET formation, NOX2, p47phox
			March et al. 2006	CS‐induced COPD model	A/J mice	EGCG was administered via drinking water at a dose of 50 μg/mL.	Anti‐inflammatory	↓: number of inflammatory cells, lactate dehydrogenase
			Chan et al. 2012	CS‐induced COPD model	Sprague ‐Dawley rats	A 10 mL dose of Lung Chen tea was administered daily via oral tube feeding for 56 days.	Antioxidant	↓: MDA, neutrophil elastase, MMP‐12
Flavones	Baicalin	C_15_H_10_O_5_	Wang, Mohammadtursun, et al. 2018	CS‐induced COPD model	Sprague–Dawley rats	BIA was administered via gavage at doses of 40, 80 or 160 mg/kg/d, respectively.	Anti‐inflammatory; antioxidant; anti‐airway remodeling	↑: IL‐10, HPA; ↓: MMP‐2, MMP‐9, MDA
			Ju et al. 2022	CS‐ and LPS‐induced COPD model	Sprague–Dawley rats	BIA (dissolved in normal saline) was administered daily at 40, 80, or 160 mg/kg/d for 3 weeks.	Anti‐inflammatory; antioxidant	↑: IL‐10, SOD, GSH; ↓: TNF‐α, IL‐1β, IL‐6, IL‐8, MDA, MYD88, p‐NF‐κ, Bp65/*NF‐κ*Bp65, TLR2, TLR4
			Zhang, Liu, et al. 2021	CS‐induced COPD model	Sprague–Dawley rats	BIA was administered via intragastric administration at doses of 40, 80, or 160 mg/kg.	Anti‐inflammatory	↑: HDAC2; ↓: NF‐κB, PAI‐1
	Luteolin	C_15_H_10_O_6_	Zhou et al. 2023	CS‐ and LPS‐induced COPD model	C57BL/6J mice	Starting from Week 15, Lut was orally administered at doses of 50 or 100 mg/kg 1 h prior to CS exposure.	Anti‐inflammatory; antioxidant	↑: SOD, CAT, GSH, SIRT6; ↓: MDA, LDH, TRPV1, CYP2A13
			Li et al. 2023	CS‐induced COPD model	BALB/c mice	During the 75‐day CS exposure period, Lut was administered via gavage at doses of 20 or 40 mg/kg once daily.	Anti‐inflammatory; antioxidant	↑: SOD, CAT, NQO1, HO‐1; ↓: IL‐1β, IL‐6, TNF‐α, IL‐8, MDA, NOX4, p‐p65/p65, p‐IκB/IκB
			Wang, Gu, et al. 2021	CS‐ and LPS‐induced COPD model	ICR mice	After the first LPS injection, Lut was administered via intragastric administration at doses of 5, 10, or 20 mg/kg once daily for 6 weeks.	Anti‐inflammatory	↓: EGFR, MMP9, PTGS2, MMP2, ERBB2
	Nobiletin	C_21_H_22_O_8_	Lu et al. 2022	CSE combined with *Klebsiella pneumoniae* ‐induced COPD model	Sprague–Dawley rats	Nobiletin and Icariin were administered at 2.12 mg/kg/d (1:12.5 ratio).	Anti‐inflammatory	↓: IL‐6, IL‐1β, TNF‐α, PI3K, P‐AKT, P‐p38
Flavonols	Quercetin	C_15_H_10_O_7_	Araújo et al. 2022	CS‐induced emphysema model	C57BL/6 mice	One hour before the first CS exposure, Que was administered via orogastric gavage at a dose of 10 mg/kg/d.	Anti‐inflammatory; antioxidant	↑: SOD, CAT; ↓: IL‐13, IL‐22, Macrophage recruitment, MDA
			Zhou, Cai, et al. 2025	CS‐induced COPD model	Sprague–Dawley rats	Que was administered via gastric gavage at doses of 0.25 or 1 g/kg once daily.	anti‐airway remodeling	↓: TNF‐α, IL‐1β, Wnt5a, β‐catenin, collagen fiber deposition
			Yin et al. 2024	CS‐induced COPD model	C57BL/6 mice	Que was administered at 50 mg/kg and Que‐lipo at 0.3 mg/kg via intraperitoneal injection; Que‐lipo was administered at 0.3 mg/kg via intrathecal injection.	Anti‐inflammatory; anti‐fibrosis	↑: GSH‐Px, SOD, Bcl‐2; ↓: IL‐6, TNF‐α, MDA, MPO, caspase 3/7, TGF‐β1, Rho, ROCK
	Rutin	C_27_H_30_O_16_	Chen et al. 2020	CS‐induced COPD model	BALB/c mice	Rutin was administered at doses of 200, 300, or 400 mg/kg.	Anti‐inflammatory	↓: IL‐8, TNF‐α, Platelet‐activating factor level
	Isorhamnetin	C_16_H_12_O_7_	Xu et al. 2022	CS‐ and LPS‐induced COPD model	C57BL/6J mice	Isorhamnetin was administered via gavage at doses of 30 or 60 mg/kg/d.	Anti‐inflammatory; antioxidant	↑: Nrf2, HO‐1, SOD1/2; ↓: Keap1, number of inflammatory cells
Flavanones	Naringenin	C_15_H_12_O_5_	Liu et al. 2018	CS‐induced COPD model	BALB/c mice	Naringenin was administered via gavage at doses of 20, 40, or 80 mg/kg, 2 h prior to CS exposure.	Anti‐inflammatory	↓: IL‐8, TNF‐α, MMP‐9, NF‐κB, number of inflammatory cells
	Hesperidin	C_28_H_34_O_15_	Wang et al. 2020	Establishment of COPD model by intraperitoneal injection of CSE	C57BL/6 mice	Hesperidin was administered at doses of 25 or 50 mg/kg.	Anti‐inflammatory; antioxidant	↑: SOD, CAT, PGC‐1α, SIRT1; ↓: IL‐6, IL‐8, MDA, MPO
	Isoliquiritigenin	C_15_H_12_O_4_	Yu et al. 2018	CS‐induced COPD model	C57BL/6N mice	Isoliquiritigenin was orally administered at doses of 10, 20, or 30 mg/kg 1 h prior to CS exposure.	Anti‐inflammatory; antioxidant	↑: Nrf2, HO‐1; ↓: NF‐κB p‐p65, p‐IκBα, MPO, MDA, TNF‐α, IL‐1β, number of neutrophils and macrophages
Flavanonols	Silibinin	C_25_H_22_O_10_	Park et al. 2016	CS‐ and LPS‐induced COPD model	C57BL/6N mice	Silibinin was administered via oral gavage at doses of 20 or 40 mg/kg 1 h prior to CS exposure.	Anti‐inflammatory; antioxidant	↓: ERK, SP‐1, MUC5AC, MDA
	Dihydromyricetin	C_15_H_12_O_8_	Hou et al. 2025	CS‐ and LPS‐induced COPD model	BALB/c mice	Dihydromyricetin was administered via gavage at doses of 100 or 200 mg/kg.	Anti‐inflammatory; antioxidant; anti‐apoptosis	↑: GSH, xCT, GPx4; ↓: IL‐6, TNF‐α, MDA

## 2 Literature Search Methodology

This review adopted a narrative review approach. A systematic search was conducted in the PubMed and Web of Science databases and the ClinicalTrials.gov clinical trial database from the establishment of the databases to January 2026. The search was performed using a combination of subject terms and free terms, including “Chronic Obstructive Pulmonary Disease”, “COPD”, “Chronic Obstructive”, “natural products”, “Phytotherapy”, “Plant Extracts”, “Herbal Medicine”, “Terpenes”, “Flavonoids”, “Alkaloids”, “Marine Natural Products”, “animal‐derived active substances”, and “microbial metabolites”. The inclusion criteria were as follows: (I) study types were COPD in vitro cell models, in vivo animal models, and clinical trials; (II) intervention measures: clearly exploring the intervention effects and molecular mechanisms of natural products (from plants, microorganisms, animals, and marine organisms) on COPD; (III) high‐quality published studies. The exclusion criteria were: studies on mixtures without clearly defined active components, simple clinical observations without mechanism exploration, conference abstracts, and non‐peer‐reviewed literature. Two researchers independently screened the literature. After an initial screening based on titles and abstracts, full texts were obtained, and the final 350+ articles were selected according to the inclusion and exclusion criteria.

As a narrative review, this study does not involve statistical analysis or meta‐analysis. The conclusions are drawn from qualitative synthesis and critical evaluation of the included preclinical and clinical studies.

## 3 Plant‐Derived Natural Products

Plant natural products are generally classified into primary and secondary metabolites. Based on their chemical structures, biologically active secondary metabolites can be categorized into phenolic compounds, terpenoids, alkaloids, steroids, and polysaccharides, among others (Yang, Jiao, et al. 2018). Among these, alkaloids, glycosides, terpenoids, and flavonoids isolated from various herbs have been confirmed to be key active substances that may play therapeutic roles in COPD (Biharee et al. 2024).

### 3.1 Phenolic Compounds

**FIGURE 4:**
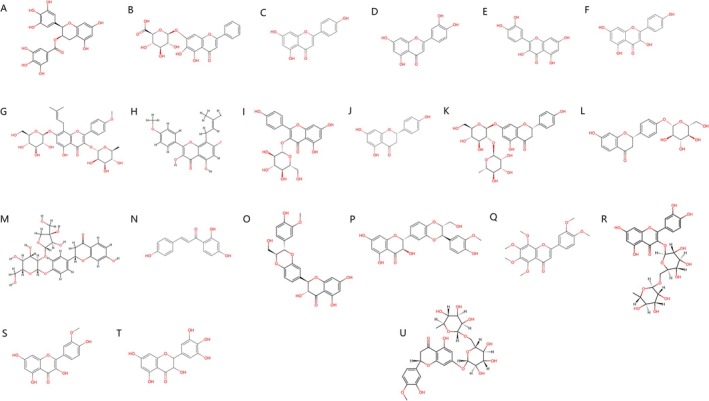
Chemical structure of natural plant‐derived flavonoids (A) Epigallocatechin‐3‐gallate, (B) Baicalin, (C) Apigenin, (D) Luteolin, (E) Quercetin, (F) Kaempferol, (G) Icariin, (H) Icaritin, (I) Astragalin, (J) Naringenin, (K) Naringin, (L) Liquiritin, (M) Liquiritin apioside, (N) Isoliquiritigenin, (O) Silymarin, (P) Silibinin, (Q) Nobiletin, (R) Rutin, (S) Isorhamnetin, (T) Dihydromyricetin, (U) Hesperidin.

**FIGURE 5:**
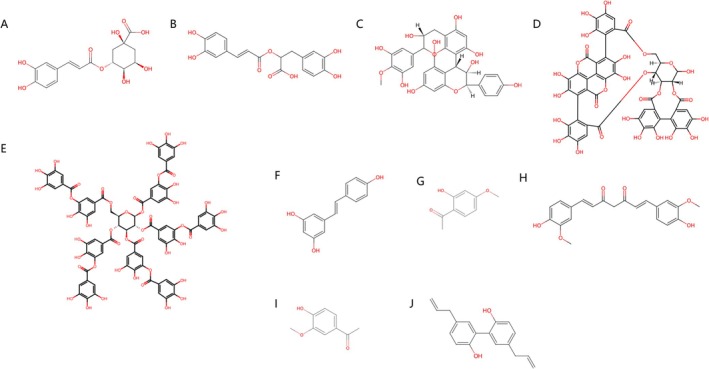
Chemical structures of natural plant‐derived phenolic acids, tannins, stilbenes, and other phenolic compounds. (A) Chlorogenic acid, (B) rosmarinic acid, (C) proanthocyanidin, (D) punicalagin, (E) tannic acid, (F) resveratrol, (G) paeonol, (H) curcumin, (I) apocynin, (J) magnolol.

Phenolic compounds represent a significant class of secondary metabolites synthesized by plants, characterized by a core structure consisting of an aromatic ring directly bonded to a hydroxyl group (‐OH) (Akyol et al. 2016). These compounds can be classified based on the number of aromatic rings, the mode of connection, and various substituents. Notable categories include flavonoids—formed by the linkage of two aromatic rings via a three‐carbon chain; phenolic acids—which feature an aromatic ring attached to both a hydroxyl group and a carboxyl group (‐COOH); stilbenes—comprising two aromatic rings connected through a trans‐ethenyl bridge; lignans—resulting from the polymerization of two phenylpropanoid molecules (C_6_‐C_3_ units); and tannins—polymerized from gallic acid or flavanols (Bolat et al. 2024; Liu, Li, and Hu 2024). Additionally, there are low‐molecular‐weight simple phenols (Chen, Lan, and Xie 2024). Figures 4 and 5 illustrate the structural formulas of these natural phenolic substances. Due to their diverse chemical structures and biological activities, phenolic compounds play crucial roles in antioxidation, anti‐inflammation, anti‐aging, and disease prevention and treatment (Del Rio et al. 2013; Ganesan and Xu 2017). Tables 1 and 2 summarize the preclinical therapeutic effects and potential mechanisms of phenolic compounds in animal models for COPD.

**TABLE 2:** Effects of tannins, stilbenes, phenolic acids, simple phenols, lignans, and other polyphenolic compounds in animal models of COPD.

Classification	Subclassification	Substance	Molecular formula	Author (s)	Animal model	Species	Dose	Effect	Mechanism
Tannins	Condensed Tannins	Proanthocyanidin	C_15_H_14_O_6_	Sul et al. 2023	Establishment of COPD model by intraperitoneal injection of CSE	BALB/c mice	Mice treated with 25, 50, or 100 mg/kg of GSPE intraperitoneally 30 min before CSE injection.	Anti‐inflammatory; anti‐autophagy	↓: TNF‐α, IL‐1β, IL‐6, ROS, NLRP3, TFEB transcription
	Hydrolysable Tannins	Tannic acid	C_76_H_52_O_46_	Rajasekar et al. 2024	Establishment of emphysema model by intratracheal instillation of elastase	C57BL/6 J mice	Tannic acid was administered via intraperitoneal injection at doses of 6.25, 12.5, or 25 mg/kg.	Anti‐inflammatory; improving emphysema	↓: TNF‐α, IFN‐γ, MCP‐1, MMP‐9, NF‐kB p65, p38 MAPK
Stilbenes	Polyhydroxystilbenes	Resveratrol	C_14_H_12_O_3_	Chen et al. 2016	CS‐ and LPS‐induced COPD model	Kunming mice	Resveratrol was administered at a dose of 50 mg/kg.	Anti‐inflammatory; anti‐fibrosis	↓: IL‐17, IL‐6, TNF‐α, TGF‐β, Beclin1
				Wang, Dong, et al. 2017	CS‐ and LPS‐induced COPD model	Wistar rats	Resveratrol was administered via gavage at a dose of 50 mg/kg.	Anti‐inflammatory; antioxidant	↑: SOD, SIRT1, PGC‐1α; ↓: IL‐6, IL‐8, MDA
				Hu et al. 2013	CS‐ and LPS‐induced COPD model	Sprague–Dawley rats	Resveratrol was administered via gavage at a dose of 25 mg/kg/d.	Anti‐left ventricular remodeling	↑: SOD, SIRT1; ↓: LVW/BW, LVEDD, LVEDP, 8‐OHdG
				Li et al. 2022	CS‐induced COPD model	C57BL/6 mice	From week 25 to week 28, RSV was administered via gavage at a dose of 200 mg/kg/d.	Anti‐inflammatory; anti‐aging	↑: HDAC2 ↓: MURF1, MAFbx, P53, P21, SMP3, L‐1β, TNF‐α
				Shi et al. 2012	CS‐induced COPD model	BALB/c mice	One hour prior to CS exposure, resveratrol (30 mg/kg) or Vam3 (a resveratrol derivative) (50 mg/kg) was administered via intragastric administration.	Antioxidant; anti‐autophagy	↑: SIRT1, FoxO3a, GSH‐Px; ↓: ROS
				Li, Sun, et al. 2014	CS‐ and LPS‐induced COPD model	Sprague–Dawley rats	Resveratrol was administered via gavage at a dose of 25 mg/kg.	Anti‐inflammatory; anti‐apoptosis	↓: CHOP, caspase‐12
				Zhang, Ding, et al. 2020	CS‐ and LPS‐induced COPD model	Sprague–Dawley rats	Resveratrol was administered via gavage at a dose of 25 mg/kg.	Antioxidant; anti‐apoptosis	↑: SIRT1, ORP150; ↓: CHOP, caspase 12, caspase 3
	Methoxystilbenes	Paeonol	C_9_H_10_O_3_	Qin et al. 2021	CS‐ and recurrent bacterial infections‐induced COPD stable phase model	Sprague–Dawley rats	Paeonol was administered via gavage at a dose of 0.25 mg/kg/d.	Anti‐mucus secretion	↓: MUC5AC, MUC5B, MUC1, MMP‐9
Phenolic acids	NA	3,4,5‐Trihydroxycinnamic acid	C_9_H_8_O_5_	Min et al. 2020	CS‐ and LPS‐induced COPD model	C57BL/6N mice	THCA was administered at doses of 20 or 40 mg/kg.	Anti‐inflammatory; antioxidant	↑: NQO1, SIRT1; ↓: IL‐6, TNF‐α, MCP‐1, MPO, neutrophil elastase activity, MAPK/NF‐κB
		Rosmarinic acid	C_18_H_16_O_8_	Yuan et al. 2025	CS‐ and LPS‐induced COPD model	BALB/cmice	Rosmarinic acid was administered via gavage at doses of 15, 30, or 60 mg/kg.	Anti‐inflammatory; anti‐apoptosis	↓: IL‐6, TNF‐α, IL‐17A, IFN‐γ, Bax/Bcl‐2, Syk, p‐Syk
Simple phenols	NA	Apocynin	C_9_H_10_O_3_	Chan et al. 2023	CS‐induced COPD model	BALB/c mice	Apocynin was administered via intraperitoneal injection at a dose of 5 mg/kg daily.	Improving vascular injury	↑: eNOS; ↓: number of macrophages, neutrophils, and lymphocytes, and airway collagen deposition
				Alateeq et al. 2024	CS‐induced COPD model	BALB/c mice	Apocynin was administered via intraperitoneal injection at a dose of 5 mg/kg/d.	Anti‐anxiety	↓: CRP, MDA, IL‐6 and IL‐1β in the amygdala
Lignans	NA	Magnolol	C_18_H_18_O_2_	Tao et al. 2025	CS combined with *Klebsiella pneumoniae* ‐induced COPD model	Sprague–Dawley rats	Magnolol was administered via gavage at doses of 25 or 50 mg/kg.	Anti‐inflammatory; antioxidant	↑: ZO‐1, E‐cadherin, PPARγ, GSH‐px, T‐SOD, TIMP‐1; ↓: TNF‐α, IL‐6, IL‐1β, MDA, MMP‐9, MMP‐12
Other polyphenol compounds	NA	Curcumin	C_21_H_20_O_6_	Yuan et al. 2018	CS‐ and LPS‐induced COPD model	Kunming mice	Curcumin was administered orally at doses of 100 or 200 mg/kg body weight for 10 consecutive days.	Anti‐inflammatory; anti‐airway remodeling	↓: IκBα, COX‐2, number of neutrophils and lymphocytes
				Zhang et al. 2017	CS‐ and LPS‐induced COPD model	Sprague–Dawley rats	Curcumin was administered via gavage at a dose of 100 mg/kg/d starting from the 31st day of modeling.	Anti‐inflammatory; antioxidant; alleviating mitochondrial damage in skeletal muscle	↑: MnSOD, GSH‐Px and CAT, PGC‐1α, SIRT3; ↓: MDA, IL‐6, TNF‐α
				Gan et al. 2016	CS‐induced COPD model	Sprague–Dawley rats	AEC II were isolated and cultured from the COPD model, and curcumin (100 μM) was administered 24 h after cultivation.	Restoring corticosteroid resistance	↑: HDAC2; ↓: MCP‐1, IL‐8, MIP‐2α, H3/H4 acetylation
				Suzuki et al. 2009	CS‐induced emphysema model	C57BL/6J mice	Curcumin was administered via oral gavage at a dose of 100 mg/kg 1 h prior to CS exposure.	Anti‐inflammatory	↓: number of neutrophils and macrophages
				Tang and Ling 2019	CS‐ and LPS‐induced COPD model	Sprague–Dawley rats	Curcumin was administered intragastrically at a dose of 150 mg/kg for 30 consecutive days.	Anti‐apoptosis	↑: SIRT1, LC3‐I, LC3‐II, Beclin1; ↓: CHOP, GRP78
				Zhang et al. 2016	CS‐ and LPS‐induced COPD model	Sprague–Dawley rats	Curcumin was administered via gavage at a dose of 100 mg/kg/d for 30 consecutive days.	Anti‐inflammatory	↓: IL‐6, IL‐8, TNF‐α, p66Shc, p‐p66Shc
				Liu, Yang, et al. 2025	PM_2.5_‐induced COPD model	BALB/cmice	Curcumin was administered daily at a dose of 100 mg/kg by gavage.	Anti‐inflammatory; antioxidant	↑: SOD, CAT, PTEN; ↓: IL‐6, IL‐1β, TNF‐α, MDA, p‐PI3K/PI3K, p‐AKT/AKT, p‐P65/P65, p‐FoxO1/FoxO1

#### 3.1.1 Flavonoids

Flavonoids represent the most diverse group of phenolic compounds and are extensively distributed across various plant species. They primarily exist in the form of glycosides, with a smaller proportion occurring as free aglycones (Dias et al. 2021). The fundamental structure of flavonoids consists of 15 carbon atoms, characterized by a diphenylpropane backbone (C_6_‐C_3_‐C_6_) that includes two aromatic rings—designated as the A ring and B ring—connected by a central three‐carbon bridge (Heleno et al. 2015; Liu, Li, and Hu 2024). Based on the position at which the B ring is attached, the degree of oxidation in the three‐carbon central chain, and whether or not cyclization occurs, flavonoids can be categorized into seven principal subcategories: flavan‐3‐ols, flavones, flavonols, flavanones, flavanonols, isoflavones, and anthocyanins (Table 1) (Šamec et al. 2021).

##### 3.1.1.1 Epigallocatechin‐3‐Gallate (EGCG)

A clinical cross‐sectional study involving 13,570 Korean participants aged 40 and older demonstrated that individuals who consumed green tea at least twice daily exhibited a significantly lower risk of COPD compared to those who did not consume green tea (OR: 0.39; 95% CI: 0.26, 0.58) (Oh et al. 2018). In a clinical study, green tea ( *Camellia sinensis* ) consumption by patients with stable COPD was found to significantly decrease serum matrix metalloproteinase 9 (MMP‐9) levels, enhance forced expiratory volume in 1 s (FEV_1_), and relieve clinical symptoms (Apriningsih et al. 2018). EGCG, a bioactive component in green tea, has been demonstrated in vitro to suppress the activation of nuclear factor kappa‐B (NF‐κB) in airway epithelial cells induced by cigarette smoke (CS), consequently decreasing pro‐inflammatory cytokines (Syed et al. 2007); concurrently, it lowered the levels of reactive oxygen species (ROS) and 4‐hydroxynonenal (4‐HNE), alleviating oxidative damage (Lakshmi et al. 2020). EGCG preclinically diminished airway inflammation, mucus hypersecretion, and oxidative damage in animal models of COPD by inhibiting epidermal growth factor receptor (EGFR) levels as well as mucin 5 AC (MUC5AC) expression, along with the NOX2/p47phox‐ROS pathway (Chan et al. 2012; Cheng, Yang, et al. 2025; Liang et al. 2017; March et al. 2006) (Table 1).

##### 3.1.1.2 Baicalin (BIA)

BIA is a flavonoid glycoside derived from the root of *Scutellaria baicalensis* Georgi (Huangqin). In CS‐induced COPD animal models, BIA demonstrates dose‐dependent, multi‐target anti‐inflammatory activity in preclinical settings centered on histone deacetylase 2 (HDAC2) upregulation (Lixuan et al. 2010). Unlike broad NF‐κB inhibitors, BIA specifically enhances HDAC2 expression, thereby suppressing inflammatory cytokines (TNF‐α, IL‐1β, IL‐6, IL‐8) and toll‐like receptor (TLR) 2/4 signaling, while concurrently reducing plasminogen activator inhibitor‐1 (PAI‐1) to protect against vascular dysfunction (Zhang, Liu, et al. 2021). This HDAC2‐centered mechanism interfaces with NF‐κB pathway modulation via deacetylation‐dependent transcriptional repression at the p65 promoter. BIA further mitigates airway remodeling through dual metalloproteinase inhibition (MMP‐2 and MMP‐9 downregulation) and antioxidant enzyme enhancement [superoxide dismutase (SOD), catalase (CAT), glutathione (GSH) activities], with concomitant malondialdehyde (MDA) reduction (Ju et al. 2022; Wang, Mohammadtursun, et al. 2018) (Table 1). At the cellular level, BIA protects against CSE‐induced apoptosis via miR‐125a suppression in human bronchial epithelial cells (Jing et al. 2024), while the HSP72‐mediated JNK pathway provides additional stress‐responsive cytoprotection (Hao et al. 2021). Collectively, BIA's mechanism highlights HDAC2 as a master epigenetic regulator that integrates anti‐inflammatory, antifibrotic, and metabolic responses in COPD pathogenesis.

##### 3.1.1.3 Apigenin

Apigenin is a flavonoid compound that is widely distributed in various plants, including *Matricaria chamomilla* (chamomile), *Apium graveolens* (celery), and *Citrus species*. In vitro studies suggest that apigenin targets oxidative stress‐induced cellular senescence, a core pathological feature of COPD (Li et al. 2021). In vitro, apigenin markedly reduces the percentage of SA‐β‐galactosidase‐positive cells by modulating the SIRT1‐NAD^+^‐CD38 axis, decreasing senescence‐related markers p16 and p21, and upregulating silent information regulator 1 (SIRT1) expression. This SIRT1‐centered anti‐senescence mechanism complements EGCG's EGFR‐centric epithelial protection and BIA's HDAC2‐centric epigenetic regulation, collectively covering multiple cell fate regulatory nodes in COPD. Additionally, apigenin inhibits mucus production and MUC5AC gene expression in airway epithelial cells via NF‐κB pathway suppression, thereby ameliorating airway inflammation and mucus hypersecretion (Seo et al. 2014).

##### 3.1.1.4 Luteolin (Lut)

Lut, a natural flavonoid compound derived from *Lonicera japonica Thunb*. (Caprifoliaceae, honeysuckle), possesses integrated redox and inflammatory modulation as well as functional mucociliary enhancement effects. Research has confirmed that Lut effectively mitigates oxidative stress and inflammatory cascades in animal models of COPD by inhibiting the NOX4/NF‐κB signaling pathway (Li et al. 2023) and through the interactive regulation of multiple pathways such as TRPV1/SIRT6 and CYP2A13/Nrf2 (Zhou et al. 2023) (Table 1). Notably, Lut specifically increased the ciliary beat frequency (CBF) in the nasal mucosa of COPD patients, enhancing the mucociliary clearance ability and improving the retention of airway secretions (Yaghi et al. 2012).

##### 3.1.1.5 Quercetin (Que)

Que is a naturally occurring flavonoid compound that is abundant in *Allium cepa* , *Malus domestica* , and *Camellia sinensis* . Preclinical studies have demonstrated that it exhibits a broad spectrum of biological activities, including antioxidation, anti‐inflammation, immune regulation, and anti‐cellular senescence (Ding et al. 2023). In animal models of emphysema, Que could reduce the levels of IL‐10, IL‐13, and IL‐22 in lung tissue, enhance the activities of SOD and CAT (Araújo et al. 2022), and inhibit the expression of MMP‐9 and MMP‐12 (Ganesan et al. 2010). It should be noted that the increase of IL‐10 and IL‐22 in this model is positively correlated with the severity of the disease. Que restores them to the baseline level, suggesting that it exerts a lung‐protective effect by inhibiting pathological inflammatory responses. Mechanistically, Que terminates the inflammatory cascade by inhibiting the NLRP3/IL‐1β inflammasome, antagonizes the fibrotic process by blocking transforming growth factor‐beta 1 (TGF‐β1) signaling, and suppresses structural remodeling by interrupting the Wnt5a/β‐catenin pathway (Araújo et al. 2022; Yin et al. 2024; Zhou, Lai, et al. 2025) (Table 1). Moreover, Que reversed oxidative damage induced by CS exposure and corticosteroid resistance through activation of the AMPK/Nrf2 axis (Mitani et al. 2017), while also upregulating genes related to epithelial regeneration such as homeobox B2 and E74‐like ETS transcription factor—thereby promoting repair mechanisms within the airway epithelium (McCluskey et al. 2024). Notably, Que's 5‐HT receptor antagonism enables MAPK pathway‐mediated mitigation of airway inflammation and peribronchiolar fibrosis (Yang et al. 2020), introducing neuro‐immune modulation absent in other flavonoids. In CS‐induced airway injury, Que blocks MUC5AC expression and EGFR phosphorylation (Yang et al. 2012), overlapping with EGCG's EGFR‐targeting yet extending to NF‐κB‐independent mucus control. Its structural analog dihydroquercetin (DHQ) further expanded the therapeutic potential for flavonoids by inhibiting ferroptosis via the Nrf2 pathway (Liu et al. 2022).

Despite its diverse pharmacological activities, Que exhibits extremely low oral bioavailability (approximately 2%), primarily attributable to extensive first‐pass metabolism, intestinal efflux transporters, and poor aqueous solubility, which collectively restrict its systemic exposure (Li et al. 2016). To overcome these obstacles, current clinical trials (Table 7) have explored different administration strategies: the NCT03989271 trial employed a high‐dose oral regimen of 2000 mg/day, while the NCT06003270 trial compared dose effects of 500 and 1000 mg/day, aiming to balance efficacy and safety through dose optimization.

##### 3.1.1.6 Kaempferol (KMF) and Its Derivatives

KMF, a flavonoid originally isolated from *Kaempferia galanga* L. and also present in *Ginkgo biloba* , *Brassica oleracea* , and *Camellia sinensis* , exerted a lung‐protective effect in preclinical models by inhibiting elastase activity (Al‐Khayri et al. 2022) and downregulating the levels of IL‐1α and C‐X‐C motif chemokine ligand 5, as identified by connectivity map analyses (Vanderstocken et al. 2018). Its important glycoside derivative, Icariin (ICA) inhibited pro‐inflammatory mediators (IL‐8 and TNF‐α), increased the anti‐inflammatory factor IL‐10 to prevent pulmonary inflammation, and reduced ROS generation to inhibit oxidative stress in animal models. The underlying mechanism might be associated with the inhibition of the NF‐κB signaling pathway, an increase in glucocorticoid receptor protein levels, and the regulation of factors related to glucocorticoid resistance (HDAC2, Nrf2) (Hu et al. 2020; Li, Sun, et al. 2014). In vitro experiments demonstrated that the hydrolysis product of ICA, Icaritin (ICT), reduced ROS levels, activated the PI3K‐Akt‐Nrf2 pathway, and upregulates GSH levels, thereby alleviating oxidative stress (Wu et al. 2014). Furthermore, another derivative of KMF, Astragalin (AST), significantly mitigated CS‐induced emphysema and pulmonary thrombosis in mice by blocking ROS production and preventing MAPK pathway activation (Kim et al. 2021).

##### 3.1.1.7 Naringenin and Naringin

Naringenin is a flavonoid compound widely found in citrus fruits and vegetables. Under experimental conditions, naringenin has been reported to protect airway ciliary structures from cigarette smoke extract (CSE) exposure through IL‐17 down‐regulation and cAMP pathway activation in cultured cells (Zhang et al. 2024). It also reduced the excessive airway mucus secretion by inhibiting the activation of EGFR and the expression of downstream factors of the PI3K/Akt signaling pathway, NF‐κB, and lowering levels of mucin MUC5AC and the generation of ROS (Yang et al. 2011). Mechanistically, in animal models, naringenin was observed to reduce pulmonary inflammation and excessive mucus production through inhibition of both the NF‐κB signaling pathway (Liu et al. 2018) (Table 1) and the EGFR‐PI3K‐Akt/ERK MAPK signal transduction pathways (Yang et al. 2011).

In vivo, its glycoside derivative, naringin, could inhibit neutrophil infiltration, reduce levels of inflammatory mediators such as IL‐8, leukotriene B4 (LTB4), and TNF‐α (Nie et al. 2012), while decreasing the activities of myeloperoxidase (MPO) and MMP‐9, increasing the contents of SOD and lipoxin A4 (Luo et al. 2012), and upregulating the expression of aquaporin‐1 (Zhang, Zhou, et al. 2022), thereby alleviating CS‐induced airway inflammation.

##### 3.1.1.8 Liquiritin (LQ) and Liquiritin Apioside (LA)

LQ and LA, both glycosylated derivatives of liquiritigenin, are naturally occurring flavonoid compounds derived from *Glycyrrhiza uralensis* Fisch. exDC. LQ could enhance the SOD activity in lung tissue and reduce MDA levels (Chen, Su, et al. 2020), and exert protective effects by modulating the nuclear receptor Nur77 (NR4A1) and the phosphorylation signaling pathway of the transcription factor c‐Jun within the activator protein‐1 (AP‐1) complex (Zhou 2020). Additionally, when combined in a 1:1 ratio with licochalcone B, LQ significantly enhanced the inhibitory effect on hematopoietic cell kinase, resulting in a synergistic antioxidant, anti‐inflammatory, and anti‐fibrotic response (Dong et al. 2024).

LA could alleviate the CS‐induced oxidative stress and inflammatory response in the lungs by inhibiting neutrophil infiltration, decreasing levels of pro‐inflammatory cytokine TNF‐α and fibrotic factor TGF‐β, and simultaneously reducing MPO levels and enhancing SOD activity (Guan et al. 2012). Importantly, animal experiments showed that LA, as a specific antitussive component, significantly reduced capsaicin‐induced cough frequency in guinea pigs at an oral dosage of 30 mg/kg (Kamei et al. 2005).

##### 3.1.1.9 Isoliquiritigenin

Liquiritigenin is a flavanone compound extracted from the roots of *Glycyrrhiza uralensis*. It exhibited antioxidant and anti‐inflammatory properties by activating the AMPK/Nrf2/ARE signaling pathway (Liu et al. 2017), inhibiting the activation of NLRP3 and NF‐κB, and downregulating the expression of inducible nitric oxide synthase (iNOS), cyclooxygenase‐2 (COX‐2), TNF‐α, and IL‐6 (Kim et al. 2008). In CS‐induced COPD mice, it suppresses inflammatory cell infiltration, reduces MPO and MDA levels, and alleviates oxidative stress and airway inflammation via the conserved Nrf2/NF‐κB signaling axis (Yu et al. 2018) (Table 1). Additionally, our previous bioinformatics analysis indicated that liquiritigenin may influence glycolysis and phagocytosis in COPD patients by regulating three key targets: AKT1, IFNG, and JUN (Huang et al. 2025). Notably, isoliquiritigenin is quickly absorbed after oral intake and can cross the blood–brain barrier. However, it breaks down easily in the body and has a short half‐life of 1–3 h. It is mainly processed by the liver through glucuronidation and sulfation, and then removed through urine (Kumar et al. 2025).

##### 3.1.1.10 Silymarin and Silibinin

Silymarin is a natural polyphenolic flavonoid extracted from the fruits and seeds of the Asteraceae plant *Silybum marianum* (Trappoliere et al. 2009). It has been shown to mitigate CS‐induced inflammatory responses by inhibiting the ERK/p38 MAPK signaling pathway and decreasing the release of pro‐inflammatory factors, including TNF‐α, IL‐6, IL‐8, and KC (Li et al. 2016). Its main active component, Silibinin, effectively inhibited airway fibrosis by suppressing CS‐induced expression of TGF‐β1 as well as Smad 2/3 phosphorylation while also reducing collagen deposition (Ko et al. 2017). In vivo, Silibinin alleviated airway inflammation and mucus secretion through inhibition of the ERK‐SP1 pathway and reduction in the expression levels of pro‐inflammatory mediators such as MUC5AC and MPO (Park et al. 2016) (Table 1).

##### 3.1.1.11 Other Flavonoid Compounds

###### 3.1.1.11.1 Nobiletin

Nobiletin is a polymethoxyflavone predominantly derived from the pericarp of *Citrus reticulata* ‘Chachi’ (Chachi mandarin). Nobiletin, a key active constituent of Bufei Yishen formula (BYF) and its modified formulas (ECC‐BYF II, ECC‐BYF III), protects the airway epithelial barrier in COPD by activating the SIRT1/AMPK/FoxO3a pathway to promote autophagy, upregulate tight junction proteins (occludin, ZO‐1, E‐cadherin), and suppress airway epithelial cell senescence (Cheng, Yan, et al. 2025; Jia et al. 2024). It also reduces mucus hypersecretion in COPD rat models via inhibiting the EGFR/PI3K/mTOR pathway (Li, Chen, et al. 2020). Furthermore, combined with ICA, nobiletin alleviates pulmonary inflammation by blocking MAPK and PI3K/Akt signaling and reducing pro‐inflammatory cytokine release (Lu et al. 2022) (Table 1).

###### 3.1.1.11.2 Rutin

Rutin, a major flavonol compound predominantly isolated from *Sophora japonica* L., effectively reduces the levels of IL‐8, TNF‐α, and platelet‐activating factor to exert anti‐inflammatory effects in vivo (Chen, Xiong, et al. 2020) (Table 1).

###### 3.1.1.11.3 Isorhamnetin

Isorhamnetin is a naturally occurring flavonol extracted from the fruits of *Hippophae rhamnoides* L. and *Ginkgo biloba L*. In CS‐induced COPD mice, it has been shown to increase the levels of Nrf2, heme oxygenase‐1 (HO‐1), and SOD1/2 in a dose‐dependent manner, while significantly downregulating the expression of Keap1, which serves as a negative feedback regulator of Nrf2 (Xu et al. 2022).

###### 3.1.1.11.4 Dihydromyricetin (DHM)

DHM, a flavanol compound derived from *Ampelopsis grossedentata*, has been shown to inhibit lipid peroxidation and pyroptosis by specifically targeting the catalytic subunit SLC7A11 (xCT) (Hou et al. 2025) (Table 1). Additionally, DHM interacted with SRC protein, leading to the upregulation of autophagy‐related proteins such as Beclin‐1, p62, and LC3B to contribute to the alleviation of mucus hypersecretion associated with COPD (Liu, Shi, et al. 2025).

###### 3.1.1.11.5 Hesperidin

Hesperidin is abundantly present in the peels of various *Citrus* species, including *Citrus aurantium* L. (bitter orange) and *Citrus reticulata* Blanco (mandarin orange), and is particularly enriched in the pericarp of these fruits. Hesperidin has been shown to mitigate the inflammatory response and oxidative stress injury in mice with COPD by upregulating the expression of peroxisome proliferator‐activated receptor gamma coactivator 1‐alpha (PGC‐1α) and SIRT1, and inhibiting the phosphorylation of NF‐κB subunit p65 (Wang et al. 2020) (Table 1).

#### 3.1.2 Phenolic Acids

The primary phenolic acids found in plants are derivatives of hydroxybenzoic acid (HBA) and hydroxycinnamic acid (HCA) (Heleno et al. 2015). HBA possesses a fundamental C_6_‐C_1_ skeleton, with representative compounds including gallic acid and vanillin, among others (Zhang, Cai, et al. 2022). In contrast, the basic structure of HCA is characterized by a C_6_‐C_3_ configuration, which primarily encompasses caffeic acid and chlorogenic acid (Heleno et al. 2015; Upadhyay and Mohan Rao 2013).

##### 3.1.2.1 Chlorogenic Acid (CGA)

*Coffea arabica* , *Lonicera japonica* , and *Eucommia ulmoides* are all natural plant sources of CGA (Lu et al. 2020). CGA selectively inhibited the PKR‐like endoplasmic reticulum kinase (PERK) and activating transcription factor 6 pathways, alleviated endoplasmic reticulum stress (ERS) to improve pulmonary fibrosis in mice (Wang, Li, et al. 2017). CGA also mitigated lipopolysaccharide (LPS)‐induced inflammatory responses and oxidative stress in RAW264.7 cells by inhibiting the NF‐κB and JNK/AP‐1 pathways (Shan et al. 2009) and activating the AMPK/PGC‐1α pathway (Gu et al. 2023).

##### 3.1.2.2 3,4,5‐Trihydroxycinnamic Acid (THCA)

THCA is a plant phenolic acid metabolite found in various species, including those from the Polygonaceae and Asteraceae families (Lopatriello et al. 2017). It could down‐regulate MAPK/NF‐κB signal transduction while simultaneously upregulating the expression of NAD(P)H:quinone oxidoreductase 1 (NQO1) and SIRT1 (Min et al. 2020). This dual action contributes to the alleviation of the inflammatory response in mice with COPD.

##### 3.1.2.3 Rosmarinic Acid (RA)

RA is commonly found in plants belonging to the Lamiaceae family, including *Rosmarinus officinalis* , *Perilla frutescens* , and *Salvia officinalis* (Ijaz et al. 2023). It inhibited airway inflammation in COPD mice induced by CS/LPS by reducing the infiltration of IL‐6, TNF‐α, IL‐17A, and IFN‐γ in BALF and lowering the Bax/Bcl‐2 ratio through the inhibition of spleen tyrosine kinase (Syk), thereby effectively suppressing cell apoptosis (Yuan et al. 2025).

#### 3.1.3 Tannins

Tannins are a class of polyphenolic compounds widely found in grains, legume seeds, fruits, and vegetables. Based on their chemical structure and properties, tannins can be mainly categorized into two groups: hydrolysable tannins (HT) and condensed tannins (CT) (Jing et al. 2022).

##### 3.1.3.1 Proanthocyanidins

CTs, commonly referred to as proanthocyanidins, are a class of condensed tannins widely distributed in plants, particularly enriched in *Vitis vinifera* L. (grape). It down‐regulated the expression of Nox4, p38 MAPK, and NF‐κB in rat lungs, and up‐regulated the level of the anti‐inflammatory factor HO‐1 to exert anti‐inflammatory effects (Ameeramja and Perumal 2018). Grape seed proanthocyanidin extract (GSPE) reduced ROS levels, decreased the oxidation of the transcription factor EB (TFEB), and inhibited its nuclear translocation, thereby blocking CSE‐induced lung inflammation and emphysema (Sul et al. 2023) (Table 2).

Oligomeric proanthocyanidins (OPC), a specific subclass of proanthocyanidins, exhibit potential therapeutic benefits in enhancing antioxidant capacity and lipid metabolism among patients with COPD. Specifically, COPD patients who ingested 150 mg of OPC orally on a daily basis for eight weeks experienced a reduction in MDA levels (*p* < 0.05), a marked increase in high‐density lipoprotein cholesterol (HDL‐C) levels (*p* < 0.05), and a decrease in the total cholesterol/high‐density lipoprotein cholesterol (TC/HDL‐C) ratio (*p* < 0.05) (Lu et al. 2018).

##### 3.1.3.2 Punicalagin (Pun)

Pun, a hydrolyzable tannin formed by the ester bond connection of gallic acid and ellagic acid, is predominantly derived from *Punica granatum* L. It suppresses macrophage inflammation and acute lung injury in mice by regulating NF‐κB and MAPK activation and downregulating FoxO3a (Cao et al. 2019) (Zeng, Zhao, et al. 2022). Pun activated the PI3K/Akt pathway and up‐regulated Nrf2‐mediated HO‐1 expression to enhance cellular antioxidant defense mechanisms (Xu, Wan, et al. 2015). In addition, previous research conducted by our group showed that Pun counteracted CSE‐induced senescence in BEAS‐2B cells by inhibiting the protease‐activated receptor PAR2/mTOR axis, reducing levels of senescence‐related markers p16 and p21, and increasing SIRT1 expression, revealing the role of pun in the regulation of cellular senescence (Xu, Kang, et al. 2024).

##### 3.1.3.3 Tannic Acid

Tannic acid, a representative hydrolysable tannin compound, has been approved by the U.S. Food and Drug Administration (FDA) as a food additive, effectively alleviates pathological progression of emphysema (Jing et al. 2022). It inhibits NF‐κB p65 and p38 MAPK phosphorylation, downregulates transcription of matrix metalloproteinases (MMPs) and apoptosis‐related genes, and reduces abnormal elastic fiber degradation products in lung tissue (Rajasekar et al. 2024) (Table 2).

#### 3.1.4 Stilbenes

The primary dietary sources of stilbene compounds include wine, peanuts, grapes, and their derivatives. These compounds represent a class of natural polyphenols characterized by a C_6_‐C_2_‐C_6_ skeleton, with their structure comprising 1,2‐diphenylethylene units (Zhou, Cai, et al. 2025). Among the isolated stilbene compounds, monomers account for about 1/4, while the remainder exists in polymeric or derivative forms (Chen, Lan, and Xie 2024). Resveratrol, as a prominent representative molecule in its monomeric form, has become a research hotspot due to its notable anti‐inflammatory, antioxidant, anti‐tumor, and cardiovascular protective effects (El Tannir et al. 2024).

##### 3.1.4.1 Resveratrol

Resveratrol is a stilbene compound predominantly isolated from the roots of Reynoutria japonica Houtt. (syn. *Polygonum cuspidatum* Sieb. et Zucc., Polygonaceae), and also found in *Vitis vinifera* L. (grape skin, seeds, and wine) and *Arachis hypogaea* L. (peanut). Previous studies conducted by our research group found that resveratrol could inhibit NF‐κB in lymphocytes of patients with COPD, leading to a reduction in the production of TNF‐α and MMP‐9, thereby exerting anti‐inflammatory and anti‐remodeling effects (Liu, Bao, et al. 2016). It could also inhibit the expression of miR‐34a, activate SIRT1 to deacetylate and inactivate NF‐κB, which subsequently weakened CSE‐induced senescence in airway epithelial cells (Zeng, Yang, and Liu 2022). In animal models of COPD, resveratrol inhibited inflammatory cytokines and oxidative stress markers (MDA and ROS), enhanced the activity of antioxidant enzymes (SOD and GSH‐Px), and down‐regulated the expression of endoplasmic reticulum stress markers (CHOP, caspase‐12 and caspase‐3), muscle atrophy‐related markers (MURF1 and MAFbx), as well as cell senescence markers (P53 and P21) (Chen et al. 2016; Hu et al. 2013; Li, Luo, et al. 2014; Shi et al. 2012; Wang, Dong, et al. 2017; Zhang, Valizadeh, et al. 2020) (Table 2). The mechanisms might involve inhibition of the NF‐κB pathway alongside activation of the SIRT1/PGC‐1α axis to achieve anti‐inflammatory and antioxidant effects; upregulation of HO‐1/Nrf2 levels for ameliorating oxidative stress damage; activation of the SIRT1–ORP150 axis for regulating ERS; upregulation of HDAC2 expression to mitigate muscle atrophy; as well as activation of Notch1 and Sirt1/FoxO3a pathways for modulating autophagy (Chen et al. 2016; Hu et al. 2013; Kode et al. 2008; Li, Luo, et al. 2014; Liu, Lin, et al. 2014; Shi et al. 2012; Wang, Dong, et al. 2017; Zhang, Valizadeh, et al. 2020). Moreover, its dimer derivative Amurensin H blocked the Syk/NF‐κB pathway to alleviate LPS/CS‐induced airway inflammation (Fan et al. 2019).

Although resveratrol is rapidly absorbed following oral administration, its bioavailability remains low due to limited solubility, extensive metabolism, and rapid systemic clearance (Kumar et al. 2025). Currently, a clinical trial (NCT03819517) is evaluating the effects of 500 mg daily resveratrol on cardiovascular health and systemic inflammation in patients with COPD, while another trial (NCT05601180) is assessing the efficacy of combined resveratrol and Que administration in respiratory diseases including COPD.

##### 3.1.4.2 Paeonol

Paeonol is a stilbene compound extracted from the root bark of *Paeonia suffruticosa* (Zhang, Cao, et al. 2019). As an active constituent of ECC‐BYF III, paeonol alleviates COPD‐related injuries through multiple mechanisms. It suppresses airway mucus hypersecretion in COPD rats by downregulating MUC5AC, MUC5B, MUC1, and MMP‐9 (Qin et al. 2021). It enhances antioxidant defense by promoting Nrf2 nuclear translocation and upregulating downstream antioxidant proteins (Xu, Shao, et al. 2025). It also attenuates inflammation by blocking the ROS‐sensitive MAPKs/NF‐κB signaling axis (Liu, Ren, et al. 2014).

#### 3.1.5 Other Polyphenol Compounds

##### 3.1.5.1 Curcumin

Curcumin, a linear diarylheptanoid compound extracted from the rhizome of *Curcuma longa* , a member of the Zingiberaceae family, has emerged as a significant natural product in COPD‐related research due to its multi‐target characteristics (Moghaddam et al. 2009). Curcumin could reduce the infiltration of inflammatory cells and the levels of pro‐inflammatory factors, COX‐2, ROS, and MDA, inhibit the expression of ERS‐related proteins (CHOP, GRP78) and pro‐fibrotic factor TGF‐β, while increasing the activity of antioxidant enzymes and the expression of autophagy‐related proteins, and up‐regulate the level of HDAC2 in COPD animal models (Gan et al. 2016; Suzuki et al. 2009; Tang and Ling 2019; Yuan et al. 2018; Zhang et al. 2016, 2017) (Table 2). Mechanistically, it might involve SIRT1 (Tang and Ling 2019), the NF‐κB/COX‐2 axis (Yuan et al. 2018), the p66Shc pathway (Zhang et al. 2016), the Nrf2‐ARE pathway (Suzuki et al. 2009), and the PGC‐1α/SIRT3 signaling pathway (Zhang et al. 2017) to exert anti‐inflammatory, antioxidant, anti‐airway remodeling, anti‐apoptotic, and anti‐skeletal muscle injury effects. Our research group further revealed that it regulated the activity of FoxO1 through the PTEN/PI3K/Akt/NF‐κB signaling axis to reverse PM_2.5_‐induced oxidative damage (Liu, Di, et al. 2025).

It is worth noting that although curcumin has multiple pharmacological activities, its clinical application is significantly limited by its extremely low bioavailability (Askarizadeh et al. 2020). Research indicates that the oral bioavailability of curcumin is less than 1%, mainly due to its poor water solubility, rapid first‐pass metabolism (glucuronidation and sulfation), and the action of intestinal efflux transporters (Aggarwal and Harikumar 2009; Hegde et al. 2023). To overcome these obstacles, researchers have developed various delivery systems, including curcumin‐loaded liposomes (Kokkinis et al. 2024; Patel et al. 2024), and nanoparticles (Chen et al. 2022) have been developed to significantly enhance preclinical therapeutic efficacy. Nanocurcumin markedly reduced IL‐6 levels (*p* < 0.001) and improved lung function indicators (FEV_1_, FVC) (Zare'I et al. 2024). Currently, two clinical trials are underway evaluating the effects of combination therapy with curcumin on sputum cytology and disease prognosis in patients with COPD (NCT01514266; NCT04687449).

##### 3.1.5.2 Apocynin

Apocynin is a naturally occurring methoxy‐substituted catechol predominantly derived from *Apocynum* species, such as *Apocynum cannabinum* and *Apocynum venetum*, and exhibits potent anti‐inflammatory and antioxidant properties. Apocynin, a selective inhibitor of the NADPH oxidase Nox2 subtype (Alateeq et al. 2024), exerts multiple protective effects by targeting Nox2‐dependent oxidative stress. It reduced lung inflammation by inhibiting inflammatory cell infiltration and the release of TNF‐α and IL‐6; improved vascular endothelial function (upregulating eNOS and downregulating 3‐NT); and inhibited platelet activation, thereby reducing the risk of cardiovascular complications (Chan et al. 2023); and alleviated anxiety‐like behaviors induced by CS, suggesting its potential as an intervention drug for mental comorbidities in COPD (Alateeq et al. 2024) (Table 2).

##### 3.1.5.3 Magnolol

Magnolol is a lignan compound extracted from the bark of *Magnolia officinalis*, a plant of the Magnoliaceae family (Lin et al. 2021). In animal experiments, it could significantly reduce pro‐inflammatory cytokines in lung tissue, increase the levels of antioxidant enzymes such as GSH and SOD, and activate the PPARγ‐SRC axis. It also upregulates the expression of tight junction proteins ZO‐1 and E‐cadherin, thus exerting anti‐inflammatory, antioxidant, and repair functions of the colonic epithelial barrier (Tao et al. 2025) (Table 2).

### 3.2 Terpenoids

**FIGURE 6:**
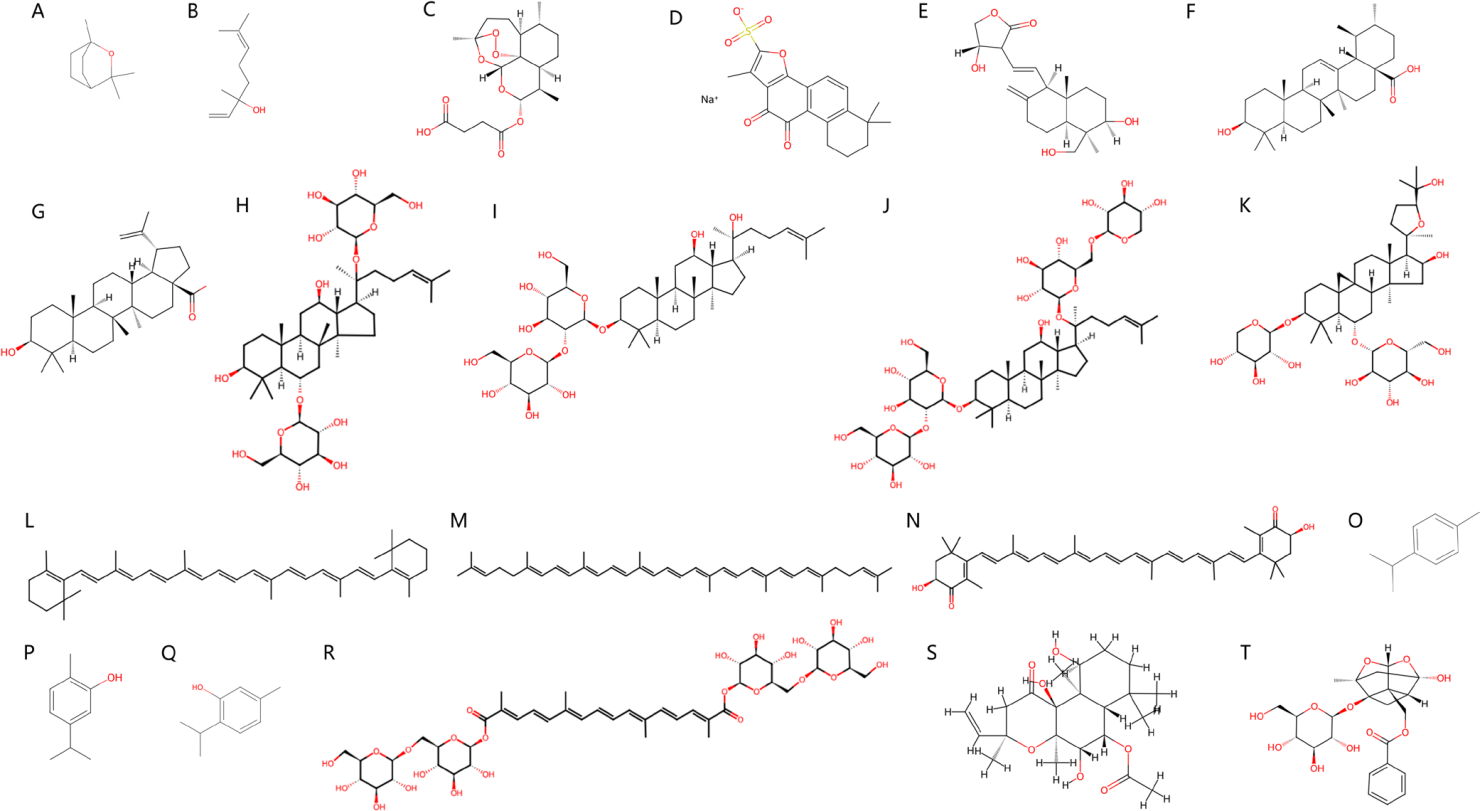
Chemical structures of natural plant‐derived terpenoids. (A) Eucalyptol, (B) Linalool, (C) Artesunate, (D) Tanshinone IIA‐sulfonic sodium, (E) Andrographolide, (F) Ursolic acid, (G) Betulinic acid, (H) Ginsenoside Rg1, (I) Ginsenoside Rg3, (J) Ginsenoside Rb3, (K) Astragaloside IV, (L) β‐Carotene, (M) Lycopene, (N) Astaxanthin, (O) *p*‐Cymene, (P) Carvacrol, (Q) Thymol, (R) Crocin, (S) Isoforskolin, (T) Paeoniflorin.

Terpenoids are the largest and most structurally diverse class of secondary metabolites in plant natural products (Zhou and Pichersky 2020), composed of isoprene units (C_5_H_8_)_n_ (Bergman et al. 2019). Based on the number of isoprene units, terpenoids can be categorized into monoterpenoids (C10), sesquiterpenoids (C15), diterpenoids (C20), triterpenoids (C30), and tetraterpenoids (C40) (Lu et al. 2023). Figure 6 shows the structural formulas of terpenoid natural substances from plants. The discovered terpenoids have properties such as antibacterial, antiviral, anti‐hyperglycemic, anti‐inflammatory, and immune regulation.

#### 3.2.1 Eucalyptol (EUC)

EUC, also known as 1,8‐cineole, is a monoterpene compound predominantly derived from *Eucalyptus globulus* Labill. It suppresses TNF‐α, IL‐1β, IL‐6, and KC production, restores antioxidant enzyme homeostasis, optimizes the MMP‐12/TIMP‐1 ratio and TIMP‐1 expression, and facilitates the repair of CS‐induced emphysema in mice (Kennedy‐Feitosa et al. 2016). A multicenter study demonstrated that compared to the currently recommended treatment regimen involving glucocorticoids in combination with long‐acting β_2_ receptor agonists, cineole (200 mg, tid, for 6 months) could significantly reduce exacerbation rates in patients with stage III/IV COPD (*p* < 0.036) (Worth et al. 2009).

#### 3.2.2 Linalool

Linalool, a natural acyclic monoterpene alcohol isolated from the essential oil of *Cinnamomum camphora* (L.) Presl var. l*inaloolifera*, possesses both anti‐inflammatory and antibacterial properties. In CS‐induced inflammatory animal models, it significantly reduces the expression of TNF‐α, IL‐6, IL‐1β, and MCP‐1 by inhibiting the NF‐κB signaling pathway (Ma et al. 2015). It also inhibited the formation of biofilms of *Acinetobacter baumannii* (Alves et al. 2016), and in combination with antibiotics, it had a synergistic bactericidal effect against methicillin‐resistant *Staphylococcus aureus* , *Pseudomonas aeruginosa* , and *Escherichia coli* , notably decreasing the minimum inhibitory concentration (MIC) values of antibiotics and reversing drug resistance (Aelenei et al. 2019), with an MIC range of 2–8 μL/mL (Alves et al. 2016). Additionally, administration of 50 mg/kg linalool modulated nicotine‐induced conditioned place preference (CPP), exhibiting effects on preventing relapse that were comparable to those observed with 2 mg/kg varenicline in male mice (*p* < 0.05) (Yunusoğlu 2021).

#### 3.2.3 Artesunate

Artesunate, a semi‐synthetic derivative of artemisinin predominantly sourced from *Artemisia annua* L., possesses anti‐inflammatory, antioxidant, and anti‐fibrotic properties in animal models. In animal experiments, it could alleviate CS‐induced inflammation and oxidative stress damage by inhibiting pro‐inflammatory signaling pathways such as PI3K/Akt and MAPK, promoting the nuclear translocation of the antioxidant transcription factor Nrf2, and reducing the levels of inflammatory factors such as IL‐8, IL‐1β, MCP‐1, and KC, as well as oxidative stress markers such as 3‐NT and 8‐hydroxy‐2′‐deoxyguanosine (8‐OHdG) (Luo et al. 2016; Ng et al. 2014). It could also significantly improve CS‐induced airway remodeling in rats by modulating the PPAR‐γ/TGF‐β1/Smad2/3 signaling pathway, inhibiting the expression of α‐smooth muscle actin (α‐SMA) and Cyclin D1 (Pan et al. 2021) (Table 3).

**TABLE 3:** Effects of terpenoids in animal models of COPD.

Subclassification	Substance	Molecular formula	Author (s)	Animal model	Species	Dose	Effect	Mechanism
Monoterpenoids	Eucalyptol	C_10_H_8_O	Kennedy‐Feitosa et al. 2016	CS‐induced emphysema model	C57BL/6 mice	Eucalyptol was administered via inhalation at doses of 1 or 10 mg/mL.	Anti‐inflammatory; antioxidant	↑: TIMP‐1, elastin; ↓: MPO, TNF‐α, IL‐1β, IL‐6, KC, TGF‐β1, MDA
	Paeoniflorin	C_23_H_28_O_11_	Lin et al. 2016	CS‐induced COPD model	Sprague–Dawley rats	Paeoniflorin was administered via gavage at doses of 12, 24, or 48 mg/kg/d.	Antioxidant	↑: Nrf2, SOD, OH‐1, T‐AOC, γ‐GCS; ↓: ROS, MDA
	p‐Cymene	C_10_H_14_	Games et al. 2016	PPE‐induced COPD model	C57BL/6 mice	10 μL of p‐Cymene was administered via intranasal instillation.	Anti‐inflammatory; improving emphysema	↓: IL‐6, KC, IL‐1β, IL‐17, MMP‐9, 8‐iso‐PGF2α, p‐65‐NF‐κB
	Carvacrol	C_10_H_14_O				10 μL of Carvacrol was administered via intranasal instillation.		
	Thymol	C_10_H_14_O				10 μL of Thymol was administered via intranasal instillation.		
Sesquiterpenoids	Artesunate	C_19_H_28_O_8_	Pan et al. 2021	CS‐induced COPD model	Sprague–Dawley rats	Artesunate was administered via intraperitoneal injection at doses of 25, 50, or 100 mg/kg, 1 h prior to CS exposure.	Anti‐inflammatory; antioxidant; anti‐airway remodeling	↓: IL‐6, IL‐8, TNF‐α, ICAM‐1, ROS, GSH, TGF‐β1/Smad
Diterpenoids	Tanshinone	NA	Yang et al. 2023	CS‐ and LPS‐induced COPD model	C57BL/6J mice	TS was administered via inhalation using a PARI nebulizer (5 mg/kg, 30 min per administration, twice daily) prior to CS exposure.	Anti‐inflammatory; antioxidant	↑: Hemopexin; ↓: NF‐κB, ERK1/2, NLRP3
	Sodium tanshinone IIA sulfonate	C_19_H_17_NaO_6_S	Li, Su, et al. 2018	CS‐ and LPS‐induced COPD model	C57BL/6J mice	STS was administered via inhalation using a PARI nebulizer (5 mg/kg, 30 min per administration, twice daily) prior to CS exposure.	Anti‐inflammatory; antioxidant; anti‐mucus secretion	↓: Muc5AC, Muc5B, ERK1/2, NF‐κB
			Guan et al. 2018	CS‐ and LPS‐induced COPD model	C57BL/6J mice	STS was administered via inhalation using a PARI nebulizer (5 mg/kg, 30 min per administration, twice daily) prior to CS exposure.	Anti‐inflammatory; antioxidant	↓: MAPK, HIF‐1α, TNF‐α, IL‐1β, ROS, HO‐1, NOX1, p‐ERK, p38 MAPK
			Li, Sun, et al. 2020	CS‐ and LPS‐induced COPD model	C57BL/6J mice	STS was administered via intraperitoneal injection at a dose of 10 mg/kg/d on days 92, 93, 94, 95, and 96.	Anti‐inflammatory; antioxidant; anti‐mucus secretion	↓: IL‐6, KC, Muc5ac, Muc5b, ERK1/2, NF‐κB/p65
			Tian et al. 2022	CS‐ and LPS‐induced COPD model	C57 mice	STS was administered via inhalation through a PARI nebulizer (5 mg/kg, 30 min per session, twice daily) prior to CS exposure.	Protective effect	↑: miR‐486‐5p
	Andrographolide	C_20_H_30_O_5_	Yu et al. 2024	PPE and LPS‐induced AECOPD model	C57BL/6 mice	AGP was administered via intraperitoneal injection at doses of 5 or 10 mg/kg, 2 h prior to the last LPS stimulation.	Anti‐inflammatory	↓: NLRP3, CARD (PYCARD), Caspase‐1, IL‐1β, IL‐1
	Isoforskolin	C_22_H_34_O_7_	Xiao et al. 2021	CS and influenza virus‐induced AECOPD model	C57BL/6 J mice	Isoforskolin was administered orally at doses of 0.5 or 2 mg/kg.	Anti‐inflammatory	↓: TNF‐α, IL‐1β, IL‐6, IL‐17A, MCP‐1, RORγt, STAT3, Th17, IL‐17A, NLRP3, ASC, caspase‐1
	Crocin	C_44_H_64_O_24_	Xie et al. 2019	CS‐induced COPD model	C57BL/6 mice	Crocin was administered orally at a dose of 50 mg/kg 1 h prior to exposure to CS.	Anti‐inflammatory; anti‐depression	↓: IL‐1β, IL‐6, TNF‐α, p‐PI3K, p‐NF‐κBp65
Triterpenoids	Ursolic acid	C_30_H_48_O_3_	Lin et al. 2017	CS‐induced emphysema model	Wistar rats	UA was administered intragastrically at doses of 10, 20, or 40 mg/kg body weight/day prior to CS exposure.	antioxidant; anti‐apoptosis	↑: Nrf2/ARE, GSH, Bcl‐2; ↓: Bax, cleaved‐caspase3, cleaved‐caspase12, MDA 8‐OHdG
			Lin, Hou, Han, Yin, et al. 2019	CS‐induced emphysema model	Wistar rats	UA was administered via gavage at doses of 10, 20, or 40 mg/kg, 30 min prior to the first CS exposure.	antioxidant; anti‐airway remodeling; improving muscle atrophy	↓: α‐SMA, EMT, EndMT, TGF‐β1, p‐Smad2/3
			Lin, Hou, Han, Yin, et al. 2019	Establishment of emphysema model by intraperitoneal injection of CSE	Sprague–Dawley rats	UA was administered via gavage at a dose of 20 mg/kg.	Anti‐apoptosis; anti‐airway remodeling	↑: Bcl‐2; ↓: cleaved‐caspase3/9/12, p‐Smad2, p‐Smad3, UPR, PERK‐CHOP
			Li, Sun, et al. 2020	PM_2.5_‐induced COPD model	NA	Ursolic acid derivatives (LUAD) were administered at doses of 30, 50, or 100 mg/kg.	Anti‐inflammatory; antioxidant	↓: LDH, ACP, ALP, NOS, MDA, TNF‐α, IL‐6
	Betulinic acid	C_30_H_48_O_3_	Yue et al. 2021	PM_2.5_‐induced COPD model	NA	Betulinic acid was administered via the intragastric route at doses of 2, 5, or 8 mg/kg for 15 consecutive days.	Anti‐inflammatory	↑: SOD; ↓: LDH, ACP, AKP, ALB, MDA, NO, TNF‐α, IL‐6
	Ginsenoside Rg1	C_42_H_72_O_14_	Guan et al. 2017	CS‐induced COPD model	Sprague–Dawley rats	Rg1 was administered via gavage at a dose of 20 mg/kg per day, 30 min prior to CS exposure.	Anti‐fibrosis	↓: α‐SMA, collagen, MMP‐9, TGFβRI, p‐Smad2/3
			Tan et al. 2024	CS‐induced COPD model	C57BL/6 mice	Rg1 was administered via oral gavage at doses of 10 or 20 mg/kg per day for 4 weeks.	Anti‐inflammatory	↓: IL‐6, IL‐1β, TNF‐α, p‐PERK, ATF4, ROS, MDA, GSH, GPX4
			Guan et al. 2020	CS‐induced COPD model	Sprague–Dawley rats	Rg1 was administered via gavage 30 min prior to CS exposure.	Anti‐fibrosis	↓: IL‐6, TNF‐α, collagen type I, α‐SMA, TGF‐β1/Smad3
	Ginsenoside Rg3	C_42_H_72_O_13_	Guan et al. 2020	CS and NTHi‐induced AECOPD model	BALB/C mice	Starting from the 12th week of modeling, Rg3 was administered via gavage at doses of 10, 20, or 40 mg/kg once daily.	Anti‐inflammatory	↓: IL‐6, KC, granulocyte migration
			Wang et al. 2024	CS‐induced COPD model	BALB/C mice	Starting from the 12th week of modeling, Rg3 was administered at a dose of 40 mg/kg at least 2 h prior to CS exposure.	Inhibiting mitophagy	↑: SIRT1; ↓: IL‐6, KC, PINK1, PTEN
	Astragaloside IV	C_41_H_68_O_14_	Zhang et al. 2023	COPD model induced by CS or CS + LPS	C57BL/6J mice	AS‐IV was administered via intraperitoneal injection at a dose of 0.2 mL per 10 g prior to CS exposure.	Anti‐inflammatory; anti‐fibrosis	↑: E‐cadherin ↓: TNF‐α, IL‐1β, EGF, IGF, α‐SMA, COL1A1, FoxO3a
			Meiqian et al. 2018	CS‐induced COPD model	ICR mice	AS‐IV was administered via gavage at doses of 10, 20, or 40 mg/kg.	Anti‐inflammatory; antioxidant	↑: SOD; ↓: MDA, TNF‐α, IL‐6, JAK3/STAT3/NF‐κB
			Chen et al. 2025	CS‐ and LPS‐induced COPD model	C57BL/6 mice	AS‐IV was administered at doses of 10, 20, or 40 mg/kg.	Anti‐inflammatory	↑: mTOR and GSK‐3β; ↓: MPO, NF‐κB
			Zhang et al. 2023	CS‐induced COPD model	ICR mice	AS‐IV was administered via gavage at a dose of 40 mg/kg daily for 4 weeks.	Anti‐inflammatory	↓: IL‐1β, TNF‐α, IL‐6, IL‐17A, RORγt, CXCR4
	Platycodon saponins	C_57_H_92_O_28_	Xu et al. 2025	CS and sawdust‐induced COPD model	Sprague–Dawley rats	Platycodon saponins were administered orally at a dose of 45 mg/kg/d.	Anti‐inflammatory	↑: MUC2, ZO‐1, ki67; ↓: CCL20, IFN‐γ, TNF‐α, NO, MUC2, Ly6G, TLR4
Tetraterpenoids	Lycopene	C_40_H_56_	Mustra et al. 2019	CS‐induced COPD model	Ferrets	Lycopene was administered orally at doses of 2.2 or 6.6 mg/kg BW/day.	Improving emphysema	↓: cholesterol
			Campos et al. 2019	CS‐induced emphysema model	C57BL/6 mice	Lycopene was administered via orogastric gavage at doses of 25 or 50 mg/kg/d.	Anti‐inflammatory; antioxidant	↑: SOD, CAT, GSH; ↓: TNF‐α, IL‐10, IFN‐γ, MPO, nitrite
	Astaxanthin	C_40_H_52_O_4_	Ding et al. 2024	COPD model established by CS exposure combined with intraperitoneal injection of CSE	C57BL/6J mice	AXT was administered intraperitoneally at doses of 10, 50, or 100 mg/kg.	Anti‐airway remodeling	↑: E‐cadherin; ↓: α‐SMA, vimentin, fibronectin
			Kubo et al. 2019	CS‐induced COPD model	C57BL/6 mice	Mice were fed a diet containing astaxanthin at a concentration of 0.0158% (w/w).	Anti‐inflammatory; antioxidant	↑: Nrf2, HO‐1; ↓: neutrophil count
			Deng et al. 2023	Establishment of COPD model by intraperitoneal injection of CSE	C57BL/6J mice	AXT was administered by oral gavage at doses of 10, 50, or 100 mg/kg.	Anti‐inflammatory; antioxidant	↑: E‐cadherin, Nrf2, HO‐1, SIRT1, SOD, GSH; ↓: α‐SMA, TNF‐α, IL‐6, MPO, p‐p65, MMP‐9
			Mano et al. 2022	PPE‐induced emphysema model	C57BL/6J mice	From week 4 to week 12, mice were fed a diet containing 0.02% astaxanthin.	antioxidant; improving muscle atrophy	↓: ROS, 4‐HNE, BNIP3, p38

#### 3.2.4 Tanshinone

Tanshinone is a lipophilic diterpene quinone compound derived from the root and rhizome of *Salvia miltiorrhiza* Bge., primarily comprising tanshinone I, tanshinone IIA, tanshinone IIB, and other constituents, among which tanshinone IIA is the active component with a relatively high content (Jiang et al. 2019). TS mitigated the activation of the NLRP3 inflammasome by inhibiting the NF‐κB/ERK1/2 pathway and upregulated the expression of hemopexin, thereby relieving the inflammatory response and oxidative stress induced by CS/LPS (Yang, Yang, et al. 2023) (Table 3). Network pharmacology and molecular docking studies have confirmed that tanshinone IIA ameliorates pathological processes associated with inflammation, oxidative stress, and protease–antiprotease imbalance in COPD through modulation of key targets such as EGFR, CASP3, MMP‐9, SRC, and HSP90AA1 (Liu, Shi, et al. 2025).

Its water‐soluble derivative—tanshinone IIA sulfonic sodium— (Zhou et al. 2019) could inhibit the ERK/NF‐κB (Li, Wang, et al. 2018; Li, Sun, et al. 2020) and MAPK/HIF‐1α signaling pathways (Guan et al. 2018), up‐regulate miR‐486‐5p (Tian et al. 2022) (Table 3), and activate the SIRT1 pathway, exerting anti‐inflammatory, antioxidant, and anti‐apoptotic effects (Guan et al. 2021).

#### 3.2.5 Andrographolide (AGP)

AGP, a diterpenoid compound isolated from *Andrographis paniculata* (Burm. f.) Nees, demonstrates notable anti‐inflammatory, antioxidant, and anti‐apoptotic biological activities in preclinical and clinical studies (Hu et al. 2024). AGP reduces the expression of inflammasome components (NLRP3, ASC, Caspase‐1) and inflammatory mediators (IL‐1β, TNF‐α, MIP‐2) in vitro and in vivo. It also decreases matrix metalloproteinase expression and oxidative stress markers (MPO, HO‐1), enhances antioxidant activities (GSH, SOD), and regulates apoptosis (Caspase‐3/7) and autophagy markers (p62, LC3B‐II) (Li et al. 2013; Tan et al. 2018; Yang et al. 2013; Yu et al. 2024; Zhang, Ding, et al. 2020). The potential mechanisms might involve the down‐regulation of the NLRP3/ASC/Caspase‐1 complex (Yu et al. 2024), modulation of the miR‐218/NF‐κB pathway (Li et al. 2013) and the SIRT1/ERK pathway (Zhang, Ding, et al. 2020), as well as activation of both HO‐1/STAT3 (Yang et al. 2013) and Nrf2 signaling pathway (Tan et al. 2018). Moreover, AGP inhibited the PI3K/Akt/c‐Jun axis to enhance HDAC2 and Nrf2 levels, reversing glucocorticoid resistance in COPD (Liao et al. 2020).

#### 3.2.6 Ursolic Acid (UA)

UA is a natural pentacyclic triterpenoid compound that is abundantly found in apple peels, loquat leaves, and various medicinal plants (Liu et al. 2012). In animal models of COPD induced by CS or PM_2.5_, UA inhibited the epithelial‐mesenchymal transition (EMT) and apoptosis markers, reduced levels of inflammatory cytokines, lactate dehydrogenase (LDH), acid phosphatase (ACP), and α‐SMA, and lowered the expression of oxidative damage markers (8‐OHdG, MDA) (Li, Ma, et al. 2020; Lin et al. 2017; Lin, Hou, Han, Kang, and Wang 2019; Lin, Hou, Han, Yin, et al. 2019) (Table 3). The potential mechanisms underlying these effects might involve the inhibition of the TGF‐β1/Smad2/3 (Lin, Hou, Han, Kang, and Wang 2019; Lin, Hou, Han, Yin, et al. 2019), MAPK (Ma, Deng, et al. 2019), and PERK‐CHOP pathways (Lin, Hou, Han, Yin, et al. 2019), as well as the activation of the Nrf2/ARE pathway (Lin et al. 2017), exerting effects such as improving airway‐vascular remodeling, preventing atrophy of the soleus muscle, anti‐apoptosis, antioxidation, and anti‐inflammation in mice.

#### 3.2.7 Betulinic Acid (BA)

BA is a triterpenoid compound mainly found in *Betula platyphylla* Sukaczev or *Betula pendula* Roth (Betulaceae, white birch) bark (Lou et al. 2021). It could inhibit the levels of LDH, ACP, alkaline phosphatase (ALP), albumin (ALB), and cytokines, and up‐regulate SOD activity while reducing the expression of MDA and nitric oxide synthase (NOS), thereby alleviating PM_2.5_‐induced lung inflammation and injury in mice (Yue et al. 2021) (Table 3). Notably, a therapeutic dose of 8 mg/kg BA demonstrated optimal efficacy (Yue et al. 2021).

#### 3.2.8 Ginsenoside

Ginseng is primarily sourced from Panax ginseng C. A. Mey. and Panax notoginseng (Burk.) F. H. Chen. As a traditional Chinese medicine, it exerts multi‐target regulatory effects in the treatment of respiratory system diseases through its active components, ginsenoside Rg1, Rg3, and Rb3. A multicenter, randomized, double‐blind placebo‐controlled clinical trial demonstrated that ginseng could significantly improve the quality of life (QoL) scores and lung function parameters of patients with COPD (Xue et al. 2011).

Rg1 alleviated CS‐induced EMT and airway remodeling by inhibiting the TGF‐β1/Smad signaling pathway, reducing the expression of α‐SMA and vimentin, regulating the MMP‐9/TIMP‐1 balance, and upregulating E‐cadherin (Guan, Liu, et al. 2017; Guan, Xu, et al. 2017; Guan, Yu, et al. 2020). It also inhibited nuclear receptor coactivator 4 (NCOA4)‐mediated ferritin autophagy and the PERK/ATF4 axis in COPD mice, thereby improving COPD‐related ferroptosis (Tan et al. 2024) (Table 3).

Rg3 alleviates inflammation in acute exacerbation of COPD (AECOPD) mice induced by CS/Nontypeable *Haemophilus influenzae* by inhibiting the PI3K/Akt pathway and activating SIRT1, reducing levels of IL‐6 and KC, and inhibiting PINK1 and PTEN‐mediated mitochondrial autophagy (Guan, Yuan, et al. 2020; Wang et al. 2024) (Table 3).

Rb3 blocked trophoblast antigen protein 2 (TROP2) expression mediated by the p38 MAPK/NF‐κB pathway (Li, Cui, et al. 2021), antagonized the TGF‐β1/VEGF signaling pathway, and reduced excessive accumulation of extracellular matrix proteins such as collagen I, collagen III, and elastin (Wang, Chen, et al. 2018) in vitro, effectively improving CS‐induced airway inflammation and remodeling.

#### 3.2.9 Astragaloside IV (AS‐IV)

Astragalus membranaceus, primarily derived from *A. membranaceus* (Fisch.) Bge. var. *mongholicus* (Bge.) Hsiao (Fabaceae), is a tonic herb for qi deficiency, contains the active component AS‐IV, a triterpene saponin compound extracted from its roots. Both in vitro and in vivo studies showed that AS‐IV could inhibit inflammatory cell infiltration, the release of pro‐inflammatory factors, and the level of C‐X‐C motif chemokine receptor 4 (CXCR4), increase the activities of SOD and GSH, down‐regulate the expression of EMT markers (snail and vimentin) and the key protein GSDMD of pyroptosis, and improve lung injury induced by CS/CSE. The underlying mechanisms include: inhibition of the NF‐κB/MAPK (Chen et al. 2010; Hsieh et al. 2022) and JAK3/STAT3/NF‐κB pathways (Meiqian et al. 2018), activation of the mTORC1/GSK‐3β pathway (Chen et al. 2025), and down‐regulation of CXCR4 to restore Th17/Treg balance (Zhang, Wang, et al. 2023), thereby exerting anti‐inflammatory and antioxidant effects (Table 3). Furthermore, AS‐IV inhibited activation of the RAS/RAF/FoxO pathway to prevent pulmonary fibrosis (Zhang, Li, et al. 2023) and suppressed the TXNIP‐NLRP3‐GSDMD pathway to alleviate pyroptosis in lung epithelial cells (Wu et al. 2024) (Table 3).

PM_2.5_‐specific defense distinguishes AS‐IV. PM_2.5_ is a major environmental risk factor for COPD (Su et al. 2021). AS‐IV mitigates PM_2.5_‐induced injury through environmental toxicant‐adaptive mechanisms. It activates Nrf2/SLC7A11/GPX4 to inhibit ferroptosis (Wang, Wu, et al. 2022). Unlike DHQ's general Nrf2 activation, AS‐IV specifically targets particulate‐induced lipid peroxidation. It inhibits TLR4/MyD88/NF‐κB to reduce inflammation (Wu et al. 2021). It creates context‐dependent autophagy balance via dual mTOR modulation (Pei et al. 2021; Wang, Wang, et al. 2022). This differs from nobiletin's SIRT1‐dependent autophagy. It suppresses NLRP3/caspase‐1 pyroptosis (Huang et al. 2022). It regulates miR‐362‐3p/RUNX1 to block fibrosis (Tian, Zhang, et al. 2024).

#### 3.2.10 Carotenoids

Carotenoids are a class of tetraprenoid natural pigments widely present in photosynthetic organisms (such as plants and algae) and non‐photosynthetic organisms (including certain fungi and bacteria), with representative members including α‐carotene, β‐carotene, astaxanthin, and lycopene (Saini et al. 2022). Lycopene is predominantly derived from *Solanum lycopersicum* L. (tomato, Solanaceae).

Studies showed that serum levels of α‐carotene and β‐carotene in adults over the age of 40 exhibited a negative correlation with the incidence of COPD (Zheng et al. 2023), while levels of total ascorbic acid, lycopene, total carotenoids, and whole‐blood GSH in the plasma of COPD patients were significantly reduced, suggesting that the deficiency of antioxidant nutrients might exacerbate oxidative stress in COPD (Kodama et al. 2017).

As a liposoluble compound (Zhao et al. 2017), lycopene could inhibit the infiltration of inflammatory cells and cytokine levels, lower the total cholesterol level, and increase the activity of antioxidant enzymes, effectively alleviating the lung injury induced by CS, with the dose of 50 mg/kg/day showing the best protective effect in vivo experiments (Campos et al. 2019; Mustra Rakic et al. 2019) (Table 3).

#### 3.2.11 Astaxanthin (AXT)

AXT, a naturally occurring keto‐carotenoid predominantly found in *Haematococcus pluvialis* Flot. (Cheng and Eroglu 2021), demonstrated potential to mitigate lung injury induced by CS/CSE/PPE in preclinical models. It reduces inflammation and oxidative stress by inhibiting the NF‐κB/c‐Jun pathway and the DUOX1/NOX4 axis (Tang et al. 2023), and activating SIRT1 (Deng et al. 2023) and the Nrf2/HO‐1 pathway (Kubo et al. 2019), thereby decreasing pro‐inflammatory factors, ROS, and MDA levels while increasing SOD activity and GSH content. AXT also exerts anti‐fibrotic effects by blocking the PI3K/Akt pathway (Ding et al. 2024), inhibiting α‐SMA and vimentin, up‐regulating the expression of E‐cadherin, and regulating the balance of MMP‐9/TIMP‐1. Additionally, it improves muscle atrophy by activating the p38 MAPK signaling pathway (Mano et al. 2022) (Table 3).

#### 3.2.12 Other Terpene Compounds

##### 3.2.12.1 P‐Cymene, Carvacrol and Thymol

p‐Cymene, carvacrol, and thymol are structurally homologous monoterpenoids derived from plant essential oils (Bakkali et al. 2008). In COPD animal models, these compounds could all alleviate elastase‐induced emphysema by targeting the inhibition of the NF‐κB signaling pathway, down‐regulating the secretion of pro‐inflammatory factors such as IL‐6, KC, and IL‐17, and synergistically inhibiting MMP‐9‐mediated extracellular matrix degradation and 8‐iso‐PGF2α‐related oxidative stress responses (Games et al. 2016) (Table 3).

##### 3.2.12.2 Paeoniflorin

Paeoniflorin, a monoterpene glycoside predominantly derived from *Paeonia lactiflora Pall*. and *Paeonia suffruticosa Andr*., is a characteristic bioactive compound of *Paeonia* species. It can activate the Nrf2/ATF4 pathway. Unlike Que's Nrf2‐mediated corticosteroid resistance reversal or paeonol's phase II enzyme battery induction, this mechanism specifically couples oxidative stress response with endoplasmic reticulum stress adaptation. ATF4 activation promotes integrated stress response gene expression: HO‐1 for heme‐iron handling, γ‐glutamylcysteine synthetase (γ‐GCS) for glutathione synthesis, and SOD for superoxide dismutation (Lin et al. 2016) (Table 3). This ATF4‐dependent transcriptional coordination distinguishes it from AS‐IV's Nrf2/SLC7A11/GPX4 ferroptosis focus or EGCG's Nrf2‐NOX2 redox‐inflammatory coupling.

##### 3.2.12.3 Crocin

Crocetin, a natural carotenoid derivative found in *Crocus sativus* L. (saffron) stigma as its glycoside crocin, offers distinct advantages in the treatment of central nervous system disorders (Farkhondeh et al. 2018). It exerted anti‐inflammatory and antidepressant effects by inhibiting the PI3K/Akt/NF‐κB signaling pathway, reducing pro‐inflammatory cytokine levels in the hippocampus and ameliorating depressive‐like behaviors of COPD mice (Xie et al. 2019). Clinical studies showed that crocetin significantly reduced serum TNF‐α levels in COPD patients, improved lung function and 6‐min walk distance test (*p* < 0.05) to enhance exercise tolerance (Aslani et al. 2023), and also inhibited NF‐kB activity and coordinated the oxidation/antioxidation balance (Ghobadi et al. 2022).

##### 3.2.12.4 Isoforskolin

Isoforskolin is a natural diterpenoid compound derived from *Colquhounia coccinea* var. *mollis* (Schauer) Prain in China (Xiao et al. 2021). Isoforskolin targets acute exacerbation pathophysiology in a cigarette smoke‐influenza virus dual‐stimulus AECOPD model (Xiao et al. 2021). It suppresses Th17/IL‐17A‐driven neutrophilic inflammation. It concurrently inhibits NF‐κB/NLRP3 inflammasome activation (Table 3).

##### 3.2.12.5 Platycodon Saponins

The combination of platycodin and platycodon grandiflorum polysaccharides exerts a synergistic protective effect against CS/wood shavings‐induced lung inflammatory injury in COPD rats, which is mediated by the gut‐lung axis rather than redundant inhibition of the canonical NF‐κB pathway alone (Xu, Xu, et al. 2025) (Table 3). Specifically, this combination inhibits cytokine release in lung and small intestinal tissues, upregulates intestinal MUC2 and ZO‐1 protein expression to strengthen intestinal barrier function, reduces endotoxin and D‐lactic acid levels to improve intestinal permeability, and further blocks the TLR4/NF‐κB signaling cascade in lung tissue.

### 3.3 Alkaloids

**FIGURE 7:**
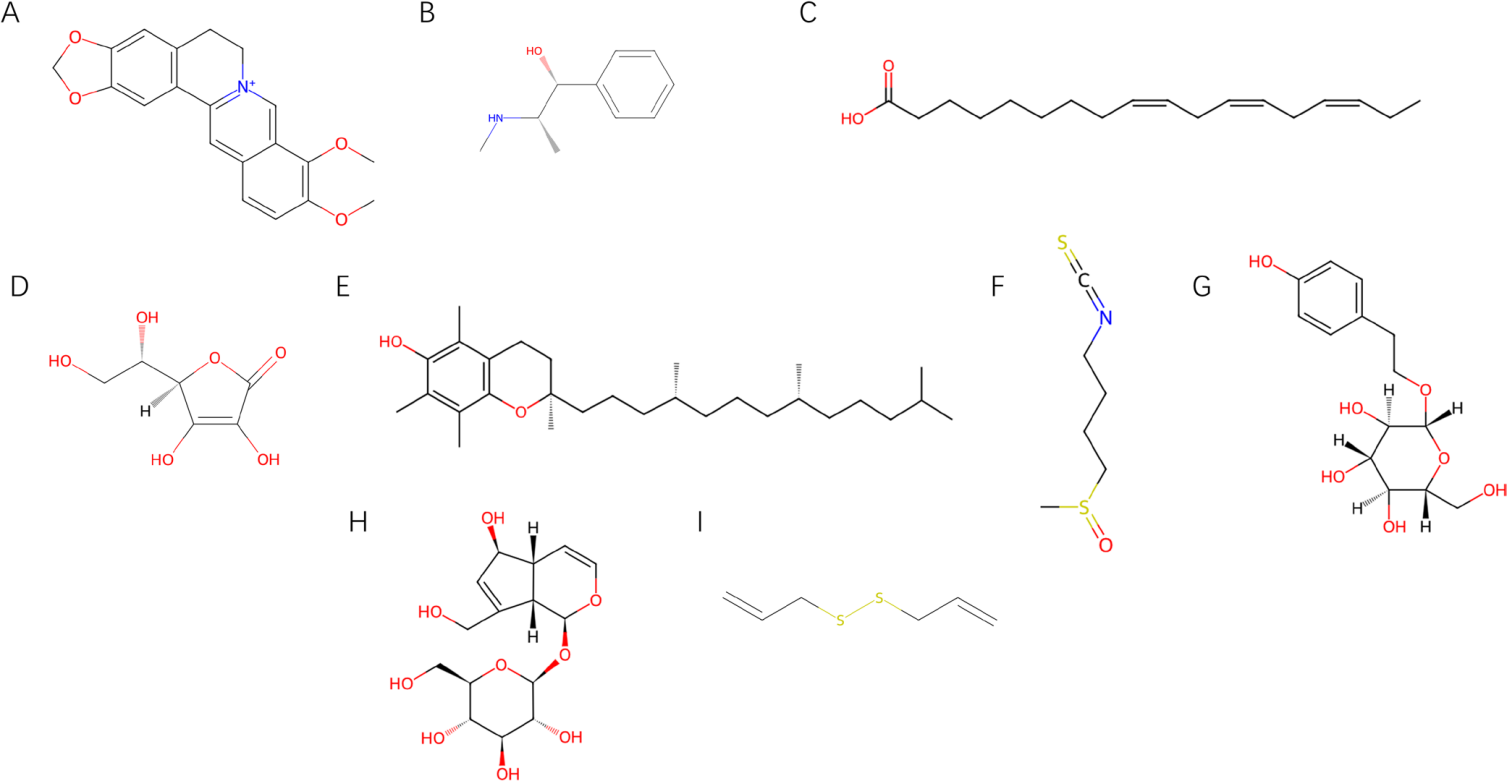
Chemical structures of natural plant‐derived alkaloids and other phytochemicals. (A) Berberine, (B) Ephedrine, (C) Alpha‐linolenic acid, (D) Vitamin C, (E) Tocopherol, (F, G) Sulforaphane, (H) Aucubin, (I) Diallyl disulfide.

Alkaloids represent a class of nitrogen‐containing organic compounds characterized by their basic properties, predominantly found in monocotyledonous plants (Oladeji et al. 2024). The structural feature of these compounds is defined by the presence of at least one nitrogen atom linked to an aromatic ring (Ma et al. 2018) (Figure 7). Alkaloids exhibit a diverse range of pharmacological activities, including anti‐inflammatory, anti‐cancer, antioxidant, and antibacterial effects (Li, Sun, et al. 2020; Ti et al. 2021).

#### 3.3.1 Berberine (Ber)

In the COPD rat model induced by CS, Bar, the principal component of *Coptis chinensis* Franch., can up‐regulate the expression of SOD, decrease MPO levels, and simultaneously inhibit the release of inflammatory cytokines to improve airway inflammation and oxidative stress injury (Liu et al. 2022) (Table 4). Both in vitro and in vivo studies have demonstrated that Ber can diminish the secretion of pro‐inflammatory factors andMUC5AC expression by inhibiting the NF‐κB pathway (Lee et al. 2007; Lin et al. 2013) the TGF‐β1/Smads signaling pathway (Wang et al. 2019), and the activation of ERK/p38 MAPKs (Xu, Li, et al. 2015), thereby improving airway inflammation and mucus hypersecretion and effectively alleviating acute lung injury induced by CS/CSE (Lin et al. 2013). Moon's research team (Moon et al. 2011) first revealed that it could inhibit the production of thymic stromal lymphopoietin (TSLP) by blocking the Caspase‐1/NF‐κB axis, opening up a new dimension in anti‐inflammation. Notably, the liposome/nanoparticle delivery system of Ber effectively overcomes its bioavailability bottleneck and can significantly enhance the therapeutic effect (Alnuqaydan et al. 2022; Liu et al. 2022; Paudel et al. 2022).

**TABLE 4:** Effects of alkaloids and other plant‐derived natural products in animal models of COPD.

Substance	Molecular formula	Author (s)	Animal model	Species	Dose	Effect	Mechanism
Berberine	C_20_H_18_NO_4_^+^	Liu et al. 2022	CS‐induced COPD model	Sprague–Dawley rats	Ber aqueous suspension (50 mg/kg) or Ber‐encapsulated SLN‐chitosan nanoparticles (containing 50 mg/kg) was administered intragastrically 1 h prior to CS exposure.	Anti‐inflammatory; antioxidant	↑: SOD; ↓: IL‐1β, IL‐6, IL‐17, TNF*α*, MPO
Ephedrine	C_10_H_15_NO	Wang, Chen, and Wu 2022	CS‐induced COPD model	C57BL/6 mice	Starting from the 7th week of CS exposure, Eph was administered via gavage at a dose of 5 mg/kg 1 h prior to CS exposure, for a duration of 6 weeks.	Anti‐inflammatory; antioxidant; anti‐apoptosis	↑: SOD, Nrf2; ↓: IL‐8, IL‐6, IL‐1β, TNF‐α, MDA, caspase‐3, p‐NF‐κB, Keap‐1, CHOP
Vitamin E	NA	Zhao et al. 2022	CS‐induced COPD model	Sprague–Dawley rats	Vitamin E was fed at 75 mg/kg daily prior to CS exposure.	Anti‐inflammatory; antioxidant	↑: SOD; ↓: MDA, COX2, STAT3
		Liu et al. 2019	CS‐ and LPS‐induced COPD model	Wistar rats	Vitamin E was formulated into a solution with a concentration of 20 mg/mL and administered via intraperitoneal injection at a dose of 40 mg/kg.	Anti‐inflammatory	↓: TNF‐α, MCP‐1, iNOS
γ‐Tocotrienol	C_28_H_42_O_2_	Peh et al. 2017	CS‐induced COPD model	BALB/c mice	γ‐Tocotrienol was administered via oral gavage at doses of 30, 100, or 250 mg/kg.	Anti‐inflammatory; antioxidant	↑: Nrf2, IL‐10; ↓: 8‐OHdG, 8‐isprostane, 3‐NT, STAT3, NF‐κB
Perilla leaf extract	NA	Yuan et al. 2022	CS‐ and LPS‐induced COPD model	BALB/c mice	PLE was administered at doses of 100, 200, or 400 mg/kg.	Anti‐inflammatory	↓: IL‐4, IL‐6, IL‐17A, IFN‐γ, TNF‐α, TLR4, Syk, PKC, NF‐κB p65
Astragalus polysaccharides	NA	Chu et al. 2016	CS‐induced COPD model	BALB/c mice	APS was administered intragastrically at a dose of 200 mg/kg from the first day to the last day of the mice's CS exposure.	Anti‐inflammatory	↓: IL‐6, IL‐8, TNF‐α
Salidroside	C_14_H_20_O_7_	Zhang, Li, and Liu 2019	CS‐induced COPD model	Wistar rats	Salidroside was administered via intraperitoneal injection at doses of 50, 100, or 200 mg/kg.	Anti‐inflammatory; improving muscle atrophy	↑: SOD, GSH, myogenin; ↓: TNF‐α, IL‐6, myostatin
Houttuynia	NA	Wang, Wu, et al. 2021	CS‐ and LPS‐induced COPD model	Sprague–Dawley rats	Houttuynia was administered via gavage at doses of 5, 10, or 25 mg/kg, 30 min prior to CS exposure.	Anti‐inflammatory; anti‐apoptosis	↓: caspase‐3, caspase‐9, TLR4, MyD88, NF‐κB (p65), IL‐6, IL‐1β, TNF‐α
Sea buckthorn extract	NA	Liu, Yang, et al. 2025	PPE and LPS‐induced COPD model	C57BL/6 mice	SBE was administered via gavage at doses of 100 or 200 mg/kg.	Anti‐inflammatory; antioxidant	↑: GPX, SLC7A11, SIRT1; ↓: p53, MAPK
Aucubin	C_15_H_22_O_9_	Liu, Li, and Hu 2024	CS‐induced COPD model	C57BL/6 mice	Aucubin was administered intragastrically at a dose of 20 mg/kg, 2 h prior to CS exposure.	Anti‐inflammatory; antioxidant; anti‐apoptosis	↑: SOD, GSH, Bcl‐2, Nrf2, HO‐1; ↓: TNF‐α, IL‐6, IL‐8, MDA, MPO, Bax
Diallyl disulfide	C_6_H_10_S_2_	Cardoso et al. 2021	CS‐induced emphysema model	C57BL/6 mice	Diallyl disulfide was administered via oral gavage at doses of 30, 60, or 90 mg/kg, three times a week.	Anti‐inflammatory; antioxidant	↑: SOD, GST; ↓: MDA, MMP12, CBR1, PNK, 4‐HNE

#### 3.3.2 Ephedrine (Eph)

Eph, the main active component of *Ephedra sinica* Stapf, is often used to treat hypotension (Fitzgerald et al. 2020), asthma (Anderson 2018), and other diseases. However, due to serious adverse reactions such as palpitations and epilepsy that Eph can cause (Haller and Benowitz 2000), the Food and Drug Administration (FDA) of the United States banned the use of dietary supplements containing Eph in 2004 (Nelson 2004). In terms of treating inflammatory response, oxidative stress, and apoptosis in COPD mice, Eph could inhibit ERS, maintain the activity of the Nrf2 antioxidant pathway, and suppress the activation of Caspase‐3 and NF‐κB (Wang, Chen, and Wu 2022) (Table 4).

### 3.4 Other Plant‐Derived Natural Products

#### 3.4.1 Polyunsaturated Fatty Acid (PUFA)

PUFA refers to fatty acids containing two or more carbon–carbon double bonds (Figure 6), which are classified into Omega‐3 and Omega‐6 based on the position of the first double bond (Broekhuizen et al. 2005). A clinical study has shown that daily supplementation of 9 g of PUFA for 8 weeks can significantly improve the exercise endurance of COPD patients (Broekhuizen et al. 2005).

Omega‐3 fatty acids primarily consist of alpha‐linolenic acid (ALA), eicosapentaenoic acid (EPA), docosapentaenoic acid (DPA), and docosahexaenoic acid (DHA) (Duvall and Levy 2016). The dietary intake of these fatty acids reduced inflammatory marker levels in COPD patients (de Batlle et al. 2012) and was inversely correlated with the risk of developing COPD (Varraso et al. 2015). As an essential fatty acid (Patchen et al. 2023), it exerted anti‐inflammatory effects by inhibiting the NF‐κB/MAPKs signaling pathway and downregulating the expression of iNOS, COX‐2, and TNF‐α (Ren and Chung 2007). Clinical data indicated that levels of ALA, EPA, and linoleic acid (LA) in the sputum from stable COPD patients were significantly lower than those observed in a smoking control group (Vander Does et al. 2019), while a cross‐sectional study in the United States confirmed that increased intake of ALA could reduce the incidence of chronic cough (*p* = 0.015) and wheezing (*p* = 0.037) in COPD patients (Lemoine et al. 2019).

LA, a representative member of Omega‐6, competes with Omega‐3 in the regulation of inflammation (Atlantis and Cochrane 2016). Its isomer, conjugated linoleic acid (CLA), has been found to decrease serum IL‐1β levels in COPD patients (Ghobadi et al. 2016), inhibit MMP‐9 and malondialdehyde levels (Matin et al. 2018), and alleviate inflammation and oxidative stress damage in COPD patients.

#### 3.4.2 Vitamin

In clinical studies, vitamin C supplementation has been shown to significantly reduce the frequency of acute exacerbations in patients with COPD (Dey et al. 2021), improve skeletal muscle mass and function (Ahmadi et al. 2020), and enhance neuromuscular fatigue tolerance (*p* < 0.05) (Hureau et al. 2021). Meta‐analyses indicated that daily supplementation with more than 400 mg of vitamin C could substantially increase antioxidant levels and improve lung function in COPD patients (Lei et al. 2022).

Vitamin E, as a fat‐soluble antioxidant, consists of 8 subtypes including 4 tocopherols and 4 tocotrienols (Peh et al. 2016). Clinical investigations have demonstrated a positive correlation between its intake and lung function (Hanson et al. 2016), as well as a negative correlation with all‐cause mortality among COPD patients (Tian, Li, et al. 2024). At the mechanistic level, vitamin E might alleviate symptoms of COPD by inhibiting the EGFR/MAPK pathway and blocking COX2‐mediated STAT3 phosphorylation and nuclear translocation (Zhao et al. 2022) (Table 4). Notably, γ‐tocotrienol significantly reduced CS‐induced airway remodeling and emphysema in mice by activating the Nrf2 antioxidant pathway and inhibiting the STAT3/NF‐κB inflammatory signal transduction, and its effect on improving lung function was superior to that of prednisolone (Peh et al. 2017).

In addition, supplementation with vitamins A and D has been found to enhance lung function in individuals with COPD (Li, Zhao, et al. 2024; Noh and Baik 2024; Van Iersel et al. 2022), whereas deficiencies in these vitamins might lead to increased morbidity and mortality related to respiratory tract conditions (Salo et al. 2022).

#### 3.4.3 Sulforaphane (SFN)

SFN is derived from *Brassica oleracea var. italica* Plenck. SFN not only restored the pathogen clearance ability via the Nrf2‐MARCO axis (Harvey et al. 2011), but also, as demonstrated in our previous research, inhibited the TLR2/4‐MyD88 pathway and reduced the production of downstream inflammatory mediators (IL‐6, TNF‐α), thereby exerting an anti‐airway inflammatory effect in COPD patients (Zeng et al. 2021).

Other natural active components in COPD animal models (Table 4): Perilla leaf extract (PLE) (Yuan et al. 2022) and Houttuynia exerted anti‐inflammatory effects by inhibiting the TLR4/Syk/PKC/NF‐κB pathway and the TLR4/MyD88/NF‐κB (p65) pathway, respectively. Notably, the therapeutic efficacy of PLE surpasses that of roflumilast and dexamethasone (Yuan et al. 2022). *Hippophae rhamnoides* extract inhibited ferroptosis by scavenging ROS, reducing NOX4 activity and down‐regulating the p53/MAPK pathway (Liu, Di, et al. 2025). Aucubin activated the Nrf2/HO‐1 antioxidant pathway (Liu, Shi, et al. 2024), and salidroside regulated the balance of myogenin and myostatin to improve skeletal muscle atrophy (Zhang, Li, and Liu 2019). Furthermore, diallyl disulfide reduced lung injury by lowering oxidative damage markers (4‐HNE, PNK, MDA) and CYP2E1 expression (Cardoso et al. 2021). Our previous study found that astragalus polysaccharides could enhance the phagocytic function of alveolar macrophages, reduce lung and systemic inflammation, and alleviate PM_2.5_‐related damage (Chu et al. 2016).

## 4 Microorganisms‐Derived Natural Products

### 4.1 Macrolides

**FIGURE 8:**
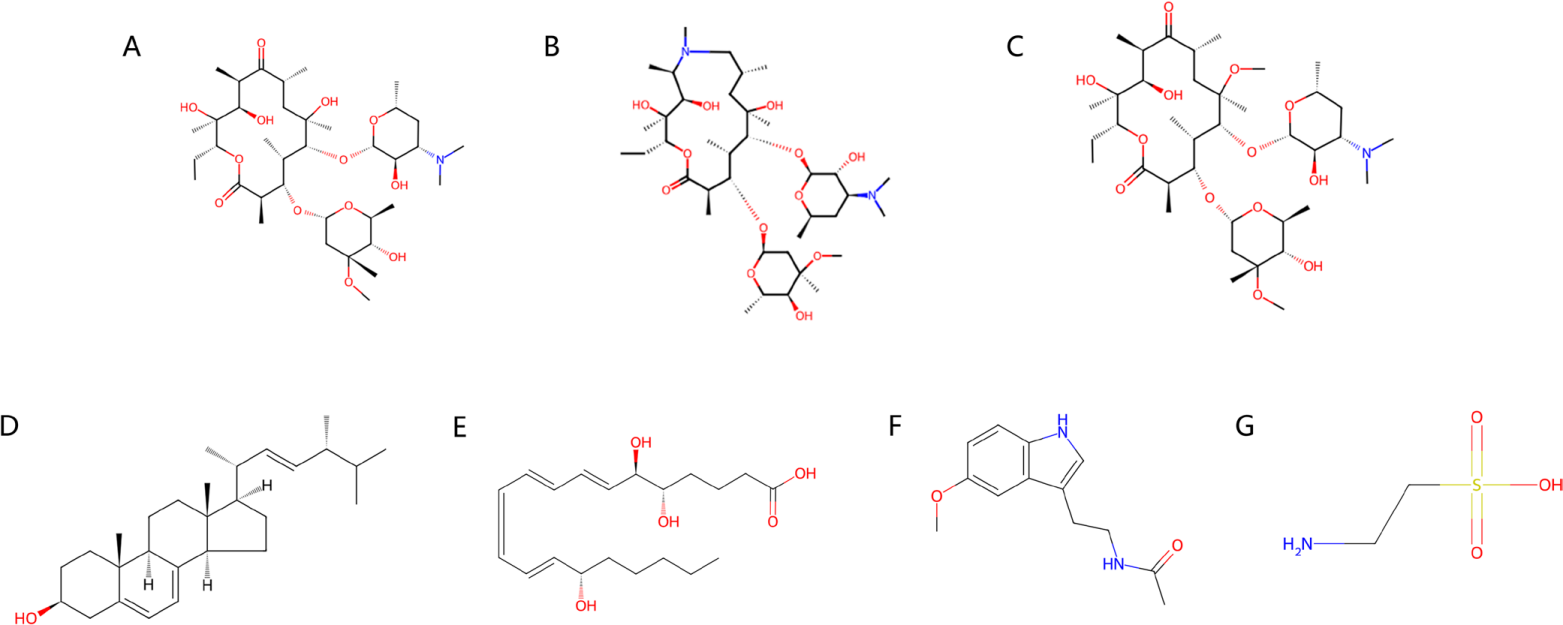
Chemical structures of microbial and animal‐derived natural products. (A) Erythromycin, (B) Azithromycin, (C) Clarithromycin, (D) Ergosterol, (E) Lipoxin A4, (F) Melatonin, (G) Taurine.

Macrolides, as secondary metabolites of Streptomyces, mainly include erythromycin (EM), and its structurally modified derivatives azithromycin (AZI) and clarithromycin (CAM) (Xiaofei et al. 2022) (Figure 8). Among them, EM is naturally produced by Streptomyces erythreus, while AZI and CAM have significantly improved oral bioavailability and reduced toxic and side effects through chemical modification. In 2017, the Global Initiative for Chronic Obstructive Lung Disease (GOLD) first included this drug in the anti‐inflammatory drug treatment plan (Zhang, Guo, et al. 2021).

A meta‐analysis indicated that long‐term macrolide treatment for more than 6 months could safely and effectively reduce the frequency of COPD exacerbations (Yao et al. 2013). Their anti‐inflammatory actions are mediated by a coordinated regulatory network rather than isolated inhibition of canonical PI3K/Akt signaling; they upregulate Nrf2 to attenuate pulmonary and systemic inflammation in COPDin vitro and in vivo (Sun, Chen, and He 2019).

EM demonstrates pleiotropic aging‐intervention: KC/TNF‐α suppression, Bcl‐2/Bax apoptosis regulation, SOD enhancement, MMP‐9/TIMP‐1 balance, and P53/P21/SA‐β‐Gal senescence marker reduction (Ma, Liu, et al. 2019; Mikura et al. 2011; Qiu et al. 2021; Xiaofei et al. 2022; Zhou et al. 2012). This multi‐dimension cellular protection exceeds single‐mechanism natural products. Mechanistically, EM activates PPARγ/NF‐κB and SIRT1‐NF‐κB axes (Ma, Liu, et al. 2019; Qiu et al. 2021), inhibits PI3K‐mTOR aging‐related signaling (Xiaofei et al. 2022), and enhances cortisol sensitivity via JNK/c‐Jun blockade (Bin et al. 2020). The derivative F528 specifically blocks NF‐κB for emphysema improvement (Zhang, Guo, et al. 2021) (Table 5).

**TABLE 5:** Effects of microorganisms‐derived natural products in animal models of COPD.

Substance	Molecular formula	Author (s)	Animal model	Species	Dose	Effect	Mechanism
Erythromycin	C_37_H_67_NO_13_	Zhou et al. 2012	CS and LPS‐induced emphysema model	Sprague–Dawley rats	Erythromycin was administered orally at a dose of 100 mg/kg, 0.5 h prior to CS exposure, for a duration of 2 weeks.	Anti‐inflammatory; anti‐apoptosis	↑: IL‐10; ↓: TNF‐α, Bax, MMP‐9, TIMP‐1, MMP‐9/TIMP‐1
		Xiaofei et al. 2022	CS‐induced COPD model	C57BL/6J mice	Erythromycin was administered via gavage at a dose of 100 mg/kg/d prior to CS exposure.	Anti‐aging	↑: SOD; ↓: P53, P21, MDA, PI3K‐mTOR
		Ma et al.^2019^	CS‐induced COPD model	BALB/c mice	Erythromycin (100 mg/kg/d) was administered intragastrically 1 h before CS exposure.		↑: SIRT1; ↓: ROS, TNF‐α, IL‐6
Erythromycin derivative, F528	NA	Zhang, Liu, et al. 2021	CS‐induced COPD model	C57BL/6J mice	F528 was administered orally at a dose of 100 mg/kg.	Anti‐inflammatory; anti‐apoptosis	↓: IL‐6, TNF‐α, Bax, MMP‐2, MMP‐9, NF‐κB p65
Clarithromycin	C_38_H_69_NO_13_	Nakanishi et al. 2009	CS‐induced COPD model	C57BL/6 mice	Clarithromycin was administered orally at doses of 25, 50, or 100 mg/kg.	Anti‐inflammatory	↓: TNF‐α, MMP‐9, number of macrophages and neutrophils
Cordyceps sinensis	NA	Yang, Jiao, et al. 2018	CS‐ and LPS‐induced COPD model	Wistar rats	Cordyceps sinensis was administered via gavage at doses of 2.5, 5, or 7.5 g/kg/day.	Anti‐inflammatory; anti‐airway remodeling	↓: α‐SMA, collagen type I, p‐Smad2, p‐Smad3, TGF‐β1
Ergosterol	C_28_H_44_O	Sun, Feng, et al. 2019	Establishment of COPD model by intraperitoneal injection of CSE	BALB/cmice	Ergosterol was administered via oral gavage at 20 or 40 mg/kg	Anti‐inflammatory; antioxidant; anti‐apoptosis	↑: SOD, CAT; ↓: MDA, NF‐κB/p65, iNOS, COX‐2, Bax
		Wang, Dong, et al. 2017	CS‐induced COPD model	ICR mice	Ergosterol was administered via gavage at doses of 20 or 50 mg/kg.	Anti‐inflammatory; antioxidant	↑: SOD, CA; ↓: MDA, TNF‐α, IL‐6, IL‐1β, JAK3/STAT3/NF‐κB
		Sun et al. 2020	Establishment of COPD model by intraperitoneal injection of CSE	Sprague–Dawley rats	Ergosterol was administered orally at doses of 2.5, 5, or 10 mg/kg.	Anti‐inflammatory; antioxidant	↑: IL‐10, TGF‐β, CD163, HDAC3; ↓: ROS, IL‐6, TNF‐α, MMP‐9, CD40, P300/CBP, PCAF, NF‐κB/p65
Lactobacillus	NA	Shen et al. 2024	PPE and LPS‐induced COPD model	BALB/cByJNarl mice	A probiotic composition (Lactobacillus GMNL‐89 and GMNL‐133 at a 1:1 ratio) was administered orally at 10^9^ CFU/0.2 mL PBS for 2 weeks.	Anti‐inflammatory	↓: IL‐6, TNF‐α, white blood cell count
*Lactobacillus rhamnosus*	NA	Carvalho et al. 2020	CS‐induced COPD model	C57BL/6 mice	Lr was administered via gavage at 1 × 10^9^ CFU/0.2 mL PBS per mouse, once daily for 7 days prior to COPD induction, with a frequency of 3 times a week.	Anti‐inflammatory; anti‐airway remodeling	↑: TIMP‐1/2, SOCS3; ↓: IL‐1, IL‐6, TNF‐α, KC, IL‐17, TGF‐β, CXCL1, MMP‐9, MMP‐12, TLR2, TLR4, TLR9, STAT3, NF‐κB
		Aimbire et al. 2019	CS‐induced COPD model	C57BL/6 mice	NA	Anti‐inflammatory	↓: MMP‐9, MMP‐12, NF‐κB, STAT3, TLR2/4/9
*Bifidobacterium breve*	NA	Aimbire et al. 2019	CS‐induced COPD model	C57BL/6 mice	NA	Anti‐inflammatory	↓: IL‐1β, IL‐6, TNF, CXCL1, CXCL8, CXCL9, CXCL10, CXCL11, KC, MMP‐9, MMP‐12, NF‐κB, STAT3, TLR2/4/9

AZI has been shown to reduce the frequency of AECOPD (Albert et al. 2011; Uzun et al. 2014) and decrease the risk of failed hospital treatment (Vermeersch et al. 2019). Its mechanism of action involved the inhibition of CS‐induced pro‐inflammatory factors and EMT markers (N‐cadherin, vimentin, α‐SMA), the up‐regulation of HDAC2 levels and key antioxidant proteins (Nrf2, SOD2) (Jain et al. 2022); the activation of the Nrf2/GCL/GSH pathway to maintain the integrity of the airway epithelial barrier (Song et al. 2023). Currently, two clinical trials are underway to evaluate the effects of AZI on COPD patients. One trial compares the preventive effects of roflumilast (Daliresp, 500 μg/day) versus AZI (250/500 μg/d, three times a week) on hospitalization or mortality events among high‐risk patients experiencing AECOPD (NCT04069312). The other focuses on stable COPD patients who have been continuously treated with AZI for at least three months; it assesses how long‐term treatment impacts disease progression in this population (NCT05772013).

CAM could preclinically inhibit macrophage accumulation and TNF‐α‐induced macrophage activation, reduce the expression of proteases such as MMP‐9 (Nakanishi et al. 2009) (Table 5), down‐regulate inflammatory factors (Nakamura et al. 2015), thereby preventing CS‐induced emphysema in mice and improving the inflammatory response.

Macrolides play a crucial protective role in the pathological processes associated with COPD through multi‐targeted regulatory mechanisms that engage various pathways.

### 4.2 Cordyceps Sinensis

Cordyceps sinensis, a traditional and valuable Chinese medicinal material, could inhibit CSE‐induced cellular senescence by blocking the ROS‐mediated PI3K/Akt/mTOR signaling pathway (Liu, Wu, et al. 2016), and improve airway remodeling in COPD rats by inhibiting the TGF‐β1/Smad signaling pathway (Yang, Wen, et al. 2018) (Table 5). Nucleosides, its key active constituent, exert anti‐inflammatory effects by activating SIRT1 to negatively regulate downstream inflammatory signaling, thereby alleviating CSE‐induced inflammation in RAW264.7 macrophages (Sun et al. 2018; Yue et al. 2013). Ergosterol, another active component, ameliorates inflammation, oxidative stress, and apoptosis in COPD models by switching macrophage polarization from pro‐inflammatory M1 to anti‐inflammatory M2 phenotype. This immunomodulatory role, together with selective suppression of downstream inflammatory cascades, accounts for its protective effects (Huan et al. 2017; Sun, Feng, et al. 2019; Sun et al. 2020) (Table 5).

### 4.3 Probiotics

Probiotics are an important component of functional foods (Champagne et al. 2018), and yogurt rich in probiotics and prebiotics is one of the main dietary sources (Hong and Luo 2024). Clinical cross‐sectional studies demonstrated that the intake of probiotics, prebiotics, or yogurt could significantly decrease the risk of COPD by 25% (Hong and Luo 2024), and the combination of budesonide and ipratropium bromide with probiotics is more conducive to controlling the pathological process of COPD (*p* < 0.05) (Chen, Wu, et al. 2024).

Animal experimental studies showed that Lactobacillus HY2782 and Bifidobacterium HY8002 could reduce the release of pro‐inflammatory factors (MCP‐1, MIP‐2) and alleviate PM2.5‐induced lung inflammation by up‐regulating Nrf2‐mediated antioxidant levels (Nam et al. 2020). *Lactobacillus acidophilus* TW01 could significantly inhibit ROS production, reduce CSE‐induced apoptosis of respiratory epithelial cells, and promote intestinal epithelial repair (Luo and Chen 2023). *Lactobacillus rhamnosus* (Lr) could inhibit lung inflammation and airway remodeling in COPD mice by coordinating the NF‐κB/STAT3/SOCS3 balance, down‐regulating the expression of TLR2/4/9, and restoring the MMP/TIMP balance (Aimbire et al. 2019; Miranda et al. 2020) (Table 5).

## 5 Animal‐Derived Natural Products

### 5.1 Lipoxin A4 (LXA4)

LXA4 is an endogenous lipid mediator synthesized from arachidonic acid by lipoxygenase and is an active molecule endogenously synthesized by animal cells through enzymatic reactions (Zhou and You 2022). Our recent research showed that LXA4 could mitigate the inflammatory response and ferroptosis in CS‐induced COPD mice by activating the ALX/FPR2 receptor, inhibiting the p38 MAPK signaling pathway, up‐regulating the SLC7A11/GPX4 axis and inhibiting iron metabolism (Li, Xu, et al. 2025) (Table 6). In addition, the LXA4 receptor agonist BML‐111 could exert anti‐inflammatory effects by inhibiting the Notch 1 signaling pathway and promoting the transformation of macrophages from pro‐inflammatory to anti‐inflammatory types (Cao et al. 2023). It could also reduce inflammation and oxidative stress in COPD mice by up‐regulating Nrf2 expression and inhibiting NLRP3 inflammasome activation (Cao et al. 2018) (Table 6).

**TABLE 6:** Effects of animal‐derived natural products in animal models of COPD.

Substance	Molecular formula	Author (s)	Animal model	Species	Dose	Effect	Mechanism
Lipoxin A4	C_20_H_32_O_5_	Li et al. 2025	CS‐induced COPD model	BALB/c mice	LXA4 was administered intraperitoneally at a dose of 10 μg/kg prior to exposure to CS.	Anti‐inflammatory; antioxidant	↑: GSH; SLC7A11; GPX4; ALX/FPR2; ↓: IL‐6, IL‐1β, TNF‐α, MDA, iron, FTH1, p38 MAPK
BML‐111	NA	Cao et al. 2018	CS‐ and LPS‐induced COPD model	Kunming mice	BML‐111 was administered intraperitoneally at doses of 1 or 10 mg/kg.	Anti‐inflammatory	↑: SOD, Nrf‐2; ↓: TGF‐β, IL‐1β, MDA, NLRP3
Melatonin	C_13_H_16_N_2_O_2_	Shin et al. 2015	CS‐ and LPS‐induced COPD model	C57BL/6N mice	Melatonin was administered intraperitoneally at doses of 10 or 20 mg/kg, 1 h prior to CS exposure.	Anti‐inflammatory; anti‐mucus secretion	↓: MUC5AC, MDA, Erk, Sp1
		Peng et al. 2018	CS‐ and LPS‐induced COPD model	Wistar rats	Melatonin was administered intraperitoneally at a dose of 10 mg/kg per day.	Anti‐inflammatory	↑: SIRT1; ↓: IL‐1, NLRP3, caspase‐1, ASC
		He et al. 2019	CS‐ and LPS‐induced COPD model	Wistar rats	Melatonin was administered intraperitoneally at a dose of 10 mg/kg per day, 1 h prior to CS exposure or LPS instillation.	Anti‐apoptosis	↑: SIRT1; ↓: caspase‐3, CHOP, caspase‐12
		Shin et al. 2020	CS‐ and LPS‐induced COPD model	C57BL/6N mice	Melatonin was administered intraperitoneally at doses of 15 or 30 mg/kg for 14 consecutive days.	Anti‐inflammatory	↑: SIRT1; ↓: p65, MMP‐9, inflammatory cell count
		Lim et al. 2021	CS‐ and LPS‐induced COPD model	C57BL/6 mice	Melatonin was administered intraperitoneally at a dose of 30 mg/kg, 1 h prior to CS exposure, for 14 consecutive days.	Anti‐inflammatory	↑: cAMP; ↓: TNF‐α, IL‐6, MMP‐9, PDE4B
		Xu et al. 2024	CS‐induced COPD model；AECOPD model induced by influenza A/H3N2 virus	C57BL/6 mice	Melatonin was administered intraperitoneally at a dose of 30 mg/kg for 7 consecutive days.	Anti‐inflammatory; anti‐apoptosis	↓: Caspase1, STAT1, iNOS, M1 polarization of pulmonary macrophages
		Shi et al. 2025	CS‐induced COPD model	BALB/c mice	Melatonin was administered intraperitoneally at a dose of 20 mg/kg daily, starting from day 61 of modeling, for 30 consecutive days.	Anti‐inflammatory; anti‐apoptosis	↓: IL‐1β, IL‐6, IL‐8, TNF‐α, Bax, c‐Caspase3, p‐PERK/PER, p‐eIF2α/eIF2α, ATF4, CHOP

**TABLE 7:** Clinical trials of natural products related to COPD.

Natural products	ID	Administration route	Dosage	Research content	Research status
Quercetin	NCT03989271	oral administration	2000 mg/day for 6 months	Effects of quercetin on inflammatory and oxidative stress biomarkers in COPD	Unknown
	NCT06003270	oral administration	500 or 1000 mg/day for 6 months	Effects of quercetin on the oxidative stress and inflammatory markers in COPD	Recruiting
Resveratrol	NCT03819517	oral administration	500 mg/day for 12 weeks	Effects of resveratrol on cardiovascular function and systemic inflammation in patients with COPD	Completed
Curcumin	NCT01514266	—	5 mg/day of Bioprine combined with 1, 1.5, or 2 g/day (of curcumin) for one month	Effects of curcumin combined with bioprine on sputum cytology in COPD patients	Completed
	NCT04687449	oral administration	950 mg/day for 90 days	Effects of curcumin on health impairment in COPD patients	Unknown
Azithromycin	NCT04069312	oral administration	250 mg/day, or 500 mg three times weekly, for 6–72 months	Effects of azithromycin on high‐risk patients with COPD exacerbation	Active, not recruiting
	NCT05772013	oral administration	according to standard prescription	Effects of azithromycin maintenance therapy on COPD exacerbations	Recruiting

**TABLE 8:** Potential assessment of natural products from different sources for COPD treatment.

Category of natural products	Main representative components	Potency assessment	Bioavailability	Clinical development stage
Plant‐derived phenolic compounds	Quercetin, Resveratrol, Curcumin, EGCG	High	Low‐Medium	Phase II clinical trials ongoing
Plant‐derived terpenoids	Eucalyptol, Ginsenoside, Astragaloside IV, Astaxanthin	High	Medium	Eucalyptol validated in Phase III clinical trials
Plant‐derived alkaloids	Berberine, Ephedrine	Medium	Low	Preclinical and early clinical stages
Other plant‐derived components	Vitamin C, Vitamin E, Sulforaphane	Medium	High	Preclinical research/Observational study stage
Microorganism‐derived natural products	Macrolides (Azithromycin, Erythromycin)	High	High	Phase III clinical validation
Animal‐derived natural products	Lipoxin A4, Melatonin, Taurine	Medium	Medium	Primarily observational clinical studies
Marine organism‐derived natural products	n‐3 PUFA, Phycocyanin	Medium	High	Nutritional intervention trials

### 5.2 Melatonin (Mel)

The neurohormone Mel is primarily synthesized and released by the pineal gland during nighttime, playing a crucial role in regulating sleep and circadian rhythms within the brain, and has pharmacological activities such as antioxidation, anti‐cancer, anti‐inflammation, and immune regulation (Hong et al. 2014; Mauriz et al. 2013; Zhang and Zhang 2014). Clinical studies showed that Melwas positively correlated with lung function (FEV_1_/FVC, FEV_1_% pred) and the activity of antioxidant enzymes (SOD, CAT, and GSH) (Gumral et al. 2009). Exogenous supplementation of Mel could significantly improve dyspnea in COPD patients (de Matos Cavalcante et al. 2012). It also enhances the exercise capacity, health status, and quality of life of patients undergoing pulmonary rehabilitation (all *p* < 0.01) (Viana et al. 2023).

Mel integrates circadian biology with cellular stress resolution through functionally coordinated mechanisms. SIRT1 activation provides metabolic resilience (Shin et al. 2020), while NLRP3 inflammasome suppression and IL‐1β production reduction dampen innate immune overactivation (Peng et al. 2018). The neurohormone uniquely modulates endoplasmic reticulum stress by attenuating CHOP and caspase‐12 expression (He et al. 2019), and further coordinates stress‐immune crosstalk through IL‐1β/STAT1 pathway blockade, thereby preventing M1 macrophage polarization and apoptosis (Xu, Li, et al. 2024). Concurrent Nrf2‐HO‐1 axis upregulation restores cellular antioxidant capacity (Mahalanobish et al. 2020), and Erk/Sp1 signaling inhibition reduces pro‐inflammatory mediators and MUC5AC‐driven mucus hypersecretion (Shin et al. 2015). This multi‐level orchestration—spanning metabolic, inflammatory, proteostatic, and redox dimensions—preclinically alleviates the pathological spectrum of COPD including inflammation, oxidative stress, mucus hypersecretion, endoplasmic reticulum stress, and apoptosis (Table 6). Additionally, our recent research has found that Mel can alleviate PM_2.5_‐induced lung injury in COPD mice by inhibiting the ERS‐related PERK/eIF2α/ATF4/CHOP pathway (Shi et al. 2025) (Table 6). This multi‐target action characteristic offers a novel strategy for precise treatment approaches for COPD.

### 5.3 Taurine

Taurine is a non‐essential sulfur‐containing amino acid that does not participate in protein synthesis. As one of the most abundant free amino acids in mammals, it is widely distributed throughout the heart, brain, liver, and muscles (Baliou et al. 2021). In vivo experiments have shown that it could reduce the inflammatory cell infiltration induced by CS, lower levels of pro‐inflammatory cytokines such as IL‐22 and IL‐6, and simultaneously increase the activities of antioxidant enzymes such as SOD and CAT to alleviate the inflammatory response and oxidative stress (de Oliveira Ramos et al. 2018). It could also improve PM‐induced emphysema in patients by comprehensively repairing the mitochondrial NADH dehydrogenase gene (Li et al. 2017).

## 6 Marine Organism‐Derived Natural Products

### 6.1 N‐3 PUFAs

Omega‐3 fatty acids, also known as n‐3 PUFAs, are abundant in fish, especially EPA and DHA. Studies showed that a high dietary intake of n‐3 PUFA (particularly EPA and DHA) and fish could dose‐dependently reduce the risk of COPD in smokers (Shahar et al. 2008), and their intake is negatively correlated with smoking behavior (Scaglia et al. 2016). Clinical intervention trials demonstrated that targeted medical nutrition supplementation (approximately 230 kcal, at least 2.0 g DHA + EPA and 10 μg 25‐hydroxyvitamin D3 per 200 mL) could significantly improve blood pressure, lipid levels (*p* < 0.05), exercise tolerance (*p* = 0.022), and dyspnea symptoms (*p* = 0.038) in COPD cachexia patients (Calder et al. 2018). Additionally, the intake of EPA, DHA, and DPA was positively correlated with FEV_1_ (Leng et al. 2017), with DHA showing the strongest correlation with a slower decline in lung function and a reduced risk of airway obstruction (Patchen et al. 2023).

### 6.2 Phycocyanin (PC)

Spirulina has been awarded the GRAS certification by the Food and Drug Administration (FDA) of the United States, being recognized as a safe food (Castro‐Gerónimo et al. 2024). PC, the most abundant PC in spirulina, not only protected the alveolar structure of COPD mice, reduced goblet cell metaplasia and collagen deposition, but also inhibited the aggregation of inflammatory cells in BALF and regulated the expression of HO‐1 and NQO1 proteins, thereby exerting anti‐inflammatory and antioxidant effects (Li, Li, et al. 2024).

## 7 Summary

**FIGURE 9:**
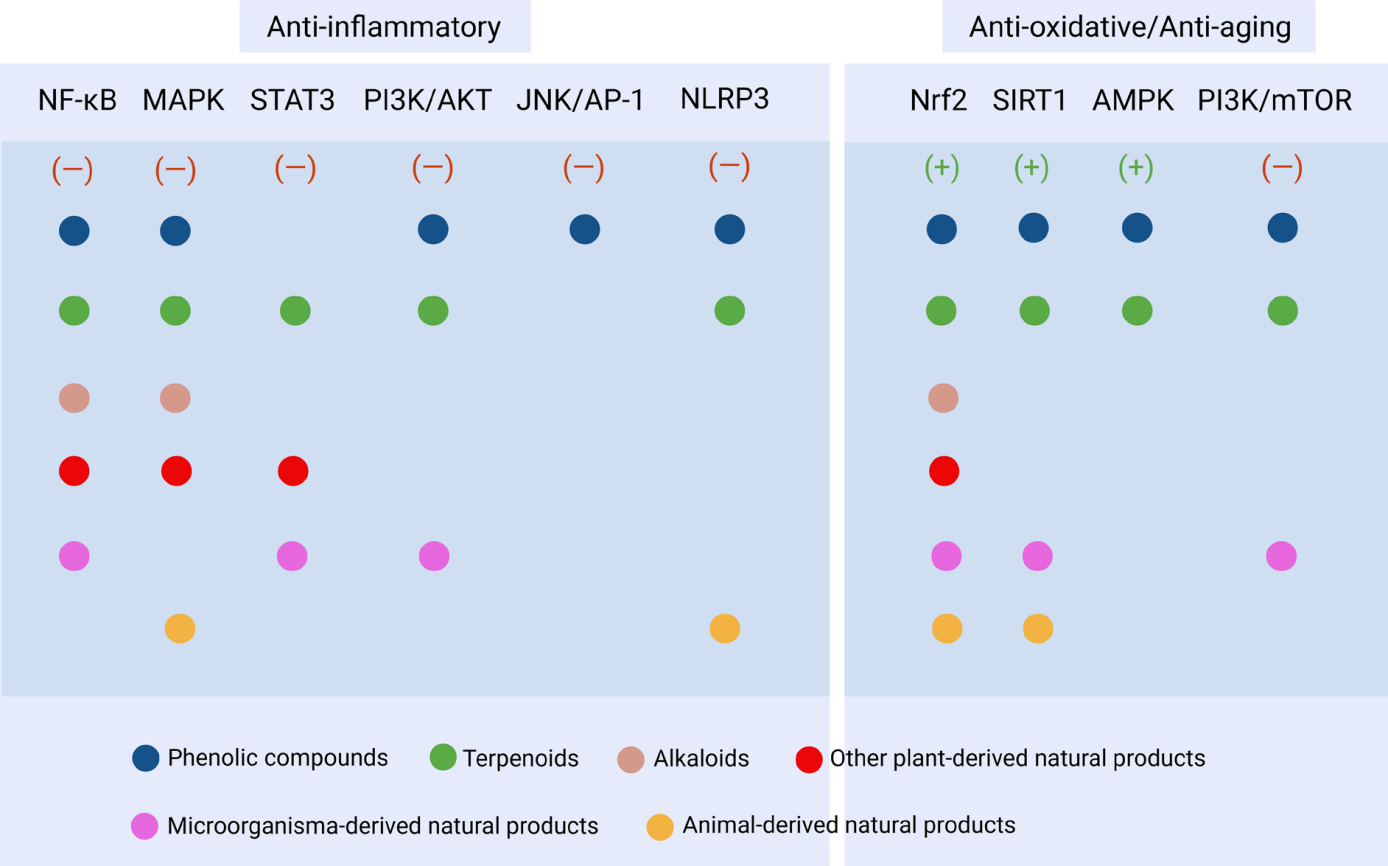
Signal pathways and cellular targets of natural products in COPD for anti‐inflammatory, anti‐oxidative, and anti‐aging effects. (Created in https://biorender.com).

**FIGURE 10:**
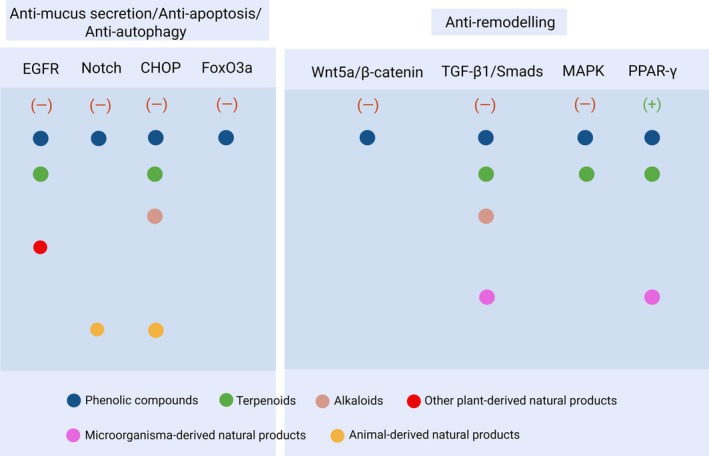
Signal pathways and cellular targets of natural products in COPD for anti‐remodeling, anti‐mucus secretion, anti‐apoptosis, and anti‐autophagy effects. (Created in https://biorender.com).

NPs have few adverse reactions and can effectively alleviate the symptoms of COPD, improve lung function, and delay the progression of the disease (Krishna et al. 2025). They have a significant advantage in the intervention during the stable period and can be used as an alternative or supplementary treatment option for COPD (Li, Liu, et al. 2025). NPs have shown potential for intervention in COPD induced by LPS/CS/CSE/PM_2.5_/PPE through various biological processes, including anti‐inflammation, antioxidation, inhibition of airway fibrosis, anti‐apoptosis, autophagy inhibition, anti‐aging effects, and the improvement of mucus hypersecretion (Figures 9 and 10).

### 7.1 Core Signal Network: Integration of Anti‐Inflammatory and Antioxidant Pathways

**FIGURE 11:**
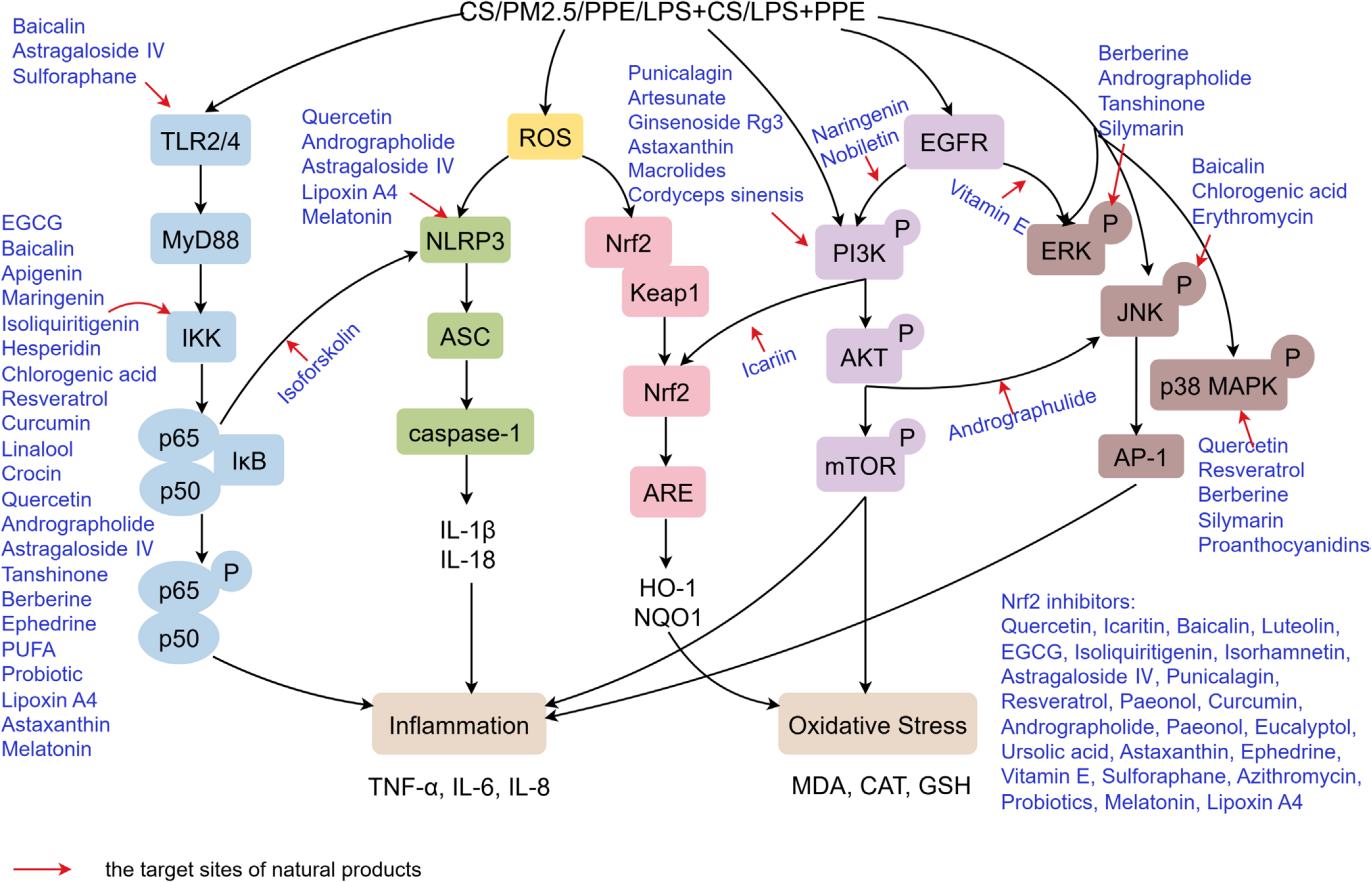
Schematic diagram of the regulatory mechanism of natural products on the main signaling pathways of inflammatory response and oxidative stress in COPD. (Created in https://www.figdraw.com).

The inhibition of the NF‐κB signaling pathway is the most consistently reported molecular mechanism in various NP studies (Figure 11). Polyphenols (such as EGCG, Que, resveratrol, curcumin), terpenoids (such as AGP, tanshinone), alkaloids (such as Ber), and animal‐derived Mel can all effectively block NF‐κB activation, thereby reducing the production of downstream pro‐inflammatory cytokines (TNF‐α, IL‐6, IL‐8). Notably, this mechanism convergence is not merely a phenotypic overlap but involves complementary regulatory nodes within the pathway. BIA and resveratrol achieve their inhibitory effects by enhancing HDAC2‐mediated deacetylation of NF‐κB p65 (Zhang, Liu, et al. 2021), while AGP acts on the upstream PI3K/Akt/c‐Jun signaling axis (Liao et al. 2020). Mel, on the other hand, reduces the production of inflammatory mediators by inhibiting the Erk/Sp1 pathway, providing a theoretical basis for combination therapy (Shin et al. 2015).

The Nrf2‐mediated antioxidant response is also widely activated in different classes of compounds, but the upstream regulatory mechanisms vary significantly (Figure 11). Polyphenols (Lut, paeonol, isoliquiritigenin) and terpenoids (EUC, AS‐IV) can consistently upregulate the expression of Nrf2 and its downstream target genes (HO‐1, NQO1, SOD), but their activation pathways are distinct. SFN activates Nrf2 by modifying Keap1 cysteine residues (Su et al. 2018), and curcumin exerts its effect through HDAC inhibition (Li, Su, et al. 2018). Additionally, probiotics from microbial sources (lactobacillus, bifidobacterium) alleviate PM_2.5_‐induced lung injury by upregulating Nrf2‐mediated antioxidant levels (Nam et al. 2020), and PC from marine sources exerts antioxidant effects by regulating the expression of HO‐1 and NQO1 (Li, Li, et al. 2024). This diversity of mechanisms across different categories in preclinical research suggests that designing combined intervention strategies may potentially produce synergistic antioxidant effects warranting further clinical investigation. This hypothesis is supported by experimental data on the combined application of LQ and licochalcone B (Dong et al. 2024).

The NF‐κB‐driven pro‐inflammatory signal and the Nrf2‐mediated antioxidant response form an inhibitory regulatory loop. Restoring the balance of this axis is a common feature of the majority of NPs. This mechanism has been reported in polyphenols (EGCG, BIA, Que, resveratrol, curcumin), terpenoids (AS‐IV, AXT), and animal‐derived products (Mel, LXA4). Existing studies consistently show that NPs can simultaneously inhibit NF‐κB activation and enhance Nrf2 nuclear translocation, thereby synergistically alleviating the two initiating factors of COPD: oxidative stress and chronic inflammation (Peh et al. 2017; Wang, Chen, and Wu 2022; Yu et al. 2018). However, the key issue of temporal dynamics remains unclear. The sequence of Nrf2 activation and NF‐κB inhibition is still not well defined in most studies, which limits the design of the optimal dosing regimen.

The MAPK signaling (ERK, p38, JNK) serves as a molecular bridge connecting oxidative stress and inflammatory responses (Figure 11). Its blockade is a common mechanism of action for tannic acid (Rajasekar et al. 2024), THCA (Min et al. 2020), paeonol (Liu, Ren, et al. 2014), and Rb3 (Li, Cui, et al. 2021). However, it is necessary to be vigilant that some compounds exhibit significant cell type‐specific effects. Resveratrol activates p38 MAPK in the process of improving muscle atrophy but inhibits this pathway in the airway inflammation model (Li et al. 2022). This directional divergence in pathway regulation highlights the decisive role of the cellular context in the effects of NPs. It also suggests that the uniform inhibition of MAPK should not be regarded as a universal therapeutic target.

Integrate the multi‐target network pharmacology framework. Different from the traditional single‐target drug concept, we propose that the action mode of NPs should be re‐examined from the perspective of network pharmacology. Multiple NPs can converge at core signaling nodes such as NF‐κB, Nrf2, and MAPK, while retaining their unique upstream regulatory mechanisms. Combined therapies targeting different entry points of the same network are expected to produce synergistic effects. Future research should be based on the principles of network pharmacology to systematically explore such theoretically grounded combined intervention strategies.

### 7.2 Emerging Mechanisms: Beyond Inflammation and Oxidative Stress

Epigenetic modifications are increasingly recognized as a unified molecular framework for the common actions of various NPs, and this has been strongly supported by cross‐category research. Polyphenols (Lut, resveratrol, curcumin, apigenin) (Hosseini et al. 2020; Li, Su, et al. 2018; Paredes‐Gonzalez et al. 2014; Zuo et al. 2018) and terpenoids (tanshinone IIA, UA) (Kim et al. 2016; Wang et al. 2014) can effectively inhibit the activities of DNA methyltransferases (DNMTs) and histone deacetylases (HDACs), thereby reversing the hypermethylation state of the Nrf2 promoter region and enhancing the histone acetylation levels in the promoter regions of antioxidant genes. Notably, the convergent regulation of HDAC2 by compounds of different structural types is particularly worthy of attention. AGP (Liao et al. 2020) and resveratrol (Huo et al. 2025) can both restore HDAC2 activity and effectively counteract glucocorticoid resistance, which is a major bottleneck in current COPD clinical treatment. The significant consistency in mechanisms among structurally diverse compounds has greatly enhanced the reliability of HDAC2 as a druggable target.

Autophagy regulation and intervention in cellular senescence represent emerging directions in NP research, but the consistency of existing evidence is relatively weak. Curcumin upregulates Beclin‐1 expression to promote autophagic flux (Jin et al. 2022); resveratrol delays cellular senescence by inhibiting miR‐34a and activating SIRT1 (Zeng, Yang, and Liu 2022); and nobiletin activates the SIRT1/AMPK/FoxO3a signaling pathway to enhance autophagy and protect epithelial barrier function (Cheng, Yan, et al. 2025). The functional effects of autophagy regulation depend on cell type and disease stage.

Ferroptosis inhibition is a recently recognized regulatory mechanism in the NP research field. DHM protects against ferroptosis by directly targeting the SLC7A11/xCT transporter (Hou et al. 2025), AS‐IV through the Nrf2/SLC7A11/GPX4 signaling axis (Wang, Wu, et al. 2022), and LXA4 by upregulating the SLC7A11/GPX4 pathway (Li, Xu, et al. 2025). Ferroptosis, as a novel therapeutic target, has opened up new avenues for the intervention of COPD. Future research should focus on developing precise delivery systems targeting ferroptosis and further explore the interactive regulatory network between ferroptosis and other programmed cell death modalities such as apoptosis, pyroptosis, and autophagy during the progression of COPD. Additionally, the gut‐lung axis, as a highly promising but underexplored therapeutic target, deserves more attention: probiotics and their metabolites may mediate systemic anti‐inflammatory effects beyond the local pulmonary microenvironment through the systemic crosstalk mechanism of the gut‐lung axis.

### 7.3 Consistency and Differences Among Compound Categories

The anti‐COPD effects of NPs exhibit significant source and chemical structure dependence. Compounds of the same category show high consistency in therapeutic effects, while structural modifications or source differences lead to variations in efficacy.

As the most thoroughly studied category of NPs, plant polyphenols demonstrate the most consistent anti‐COPD effects. Flavonoids (Que, Lut) exert anti‐inflammatory, antioxidant, and anti‐mucus hypersecretion effects by targeting the NF‐κB/Nrf2 axis (Li et al. 2023; Mitani et al. 2017; Yang et al. 2012; Zhou et al. 2023). Phenolic acids (CGA, RA), in addition to the above effects, also consistently inhibit endoplasmic reticulum stress and apoptosis (Wang, Li, et al. 2017; Yuan et al. 2025). Terpenoids, another important plant‐derived category, generally exhibit strong anti‐airway remodeling activity. Among them, triterpenoids (UA, AS‐IV) consistently exert protective effects against airway remodeling and ferroptosis by regulating the TGF‐β1/Smad and Nrf2/SLC7A11 pathways (Lin, Hou, Han, Yin, et al. 2019; Wang, Wu, et al. 2022). Microbial NPs (macrolides, Cordyceps sinensis) and animal/marine NPs (Mel, PC, n‐3 PUFA) show consistent systemic regulatory features. The former mainly improve COPD comorbidities by regulating the gut‐lung axis, while the latter enhance exercise endurance and skeletal muscle function, both going beyond simple lung‐targeted interventions.

Structural modifications of NPs lead to differences in efficacy and target selectivity. For instance, ICA (a glycoside derivative of KMF) can reverse glucocorticoid resistance by upregulating HDAC2 and GR expression, while KMF lacks this activity (Hu et al. 2020). DHQ (a hydrogenated derivative of Que) has the ability to inhibit ferroptosis by targeting the Nrf2 pathway, thereby expanding the therapeutic spectrum of flavonoids (Liu et al. 2022). The differences in effects among NPs from different sources mainly lie in the intensity of efficacy and tissue targeting. Plant‐derived NPs show stronger lung tissue‐targeting enrichment characteristics and anti‐inflammatory/antioxidant effects. Marine‐derived NPs are more effective in systemic regulation, such as improving lipid profiles and enhancing exercise endurance, making them more suitable for the intervention of COPD‐related cachexia. Probiotics, as microbial sources, specifically regulate the gut‐lung axis and are superior to other NPs in improving intestinal barrier function and reducing systemic inflammation, demonstrating source‐determined target preferences.

It is worth noting that EGCG, Que, resveratrol, curcumin, AS‐IV, Mel, and macrolides all regulate the three core signaling pathways to improve major COPD pathological processes in preclinical models. Some of them have entered the clinical research stage and are the most promising candidates for clinical translation among COPD NPs. Table 8 provides a comprehensive comparative assessment of the therapeutic potential of seven major classes of NPs from different sources for COPD, based on three core evaluation dimensions: activity potential, bioavailability, and clinical development stage. Plant‐derived terpenoids and microbe‐derived macrolides have emerged as the most promising candidates, achieving a good balance between high efficacy, acceptable bioavailability, and advanced clinical validation.

### 7.4 Key Challenges in Clinical Translation: Delivery System Optimization and Quality Control Standardization

In recent years, significant progress has been made in the development of novel drug delivery systems. Advanced formulation strategies such as nanotechnology and colloidal delivery systems have provided effective solutions for improving the oral absorption and targeted delivery of NPs. For instance, the development of curcumin‐loaded liposomes (Patel et al. 2024) and mPEG‐PLGA nanoparticles (Chen et al. 2022) has markedly enhanced its solubility and bioavailability. Clinical studies have confirmed that nano‐curcumin can effectively improve the inflammatory state and lung function indicators of patients with severe COPD (Zare'I et al. 2024). A recent study evaluating the encapsulation performance of four colloidal delivery systems for curcumin (including sodium caseinate, β‐cyclodextrin, liposome, and soy protein isolate) revealed that the sodium caseinate‐curcumin colloidal delivery system (SC‐CUR) has the best overall performance and is expected to become a high‐performance nano‐carrier in the field of functional beverages (Jin et al. 2025). Similarly, Ber encapsulated in nanoparticles (Paudel et al. 2022) and surface‐modified (such as PEGylation) can significantly increase intestinal mucosal permeability (Duong et al. 2021), effectively alleviating inflammation and oxidative stress damage induced by CS. These advanced delivery systems effectively overcome the major limitations of conventional oral administration by protecting active ingredients from gastrointestinal degradation, prolonging systemic circulation half‐life, and enhancing tissue targeting.

The variability of natural product extracts is one of the core bottlenecks restricting their clinical transformation and therapeutic stability. The chemical composition of raw plants is influenced by multiple factors such as genetic background, growth environment, harvest season, and processing and storage conditions (Zhang et al. 2012). For instance, the total flavonoid content in sea buckthorn extracts varies significantly due to different harvest latitudes and climatic conditions (Zheng et al. 2016); July is the best time to harvest sea buckthorn leaves and fruits in the Xizang region for extracting flavonoids (Yang, Yang, et al. 2023, 2023). To ensure the consistent efficacy and safety of NPs in the prevention and treatment of COPD, a standardized quality control strategy should be adopted. Firstly, advanced analytical techniques such as high‐performance liquid chromatography and gas chromatography–mass spectrometry should be used to construct characteristic chromatographic fingerprint profiles (Vander Heyden 2008), and the WHO has incorporated fingerprint technology into the guidelines for the identification and quality evaluation of medicinal plants (World Health Organization 2025). Secondly, the content range of key active components should be defined, while strictly controlling the limits of harmful impurities such as heavy metals, pesticide residues, and microbial contamination (Zhang et al. 2012). Finally, a quality management system covering the entire industrial chain should be established, including raw material procurement standards, optimization of extraction processes, monitoring of product stability, and standardized production procedures in compliance with good manufacturing practices (Ip et al. 2010).

**FIGURE 12:**
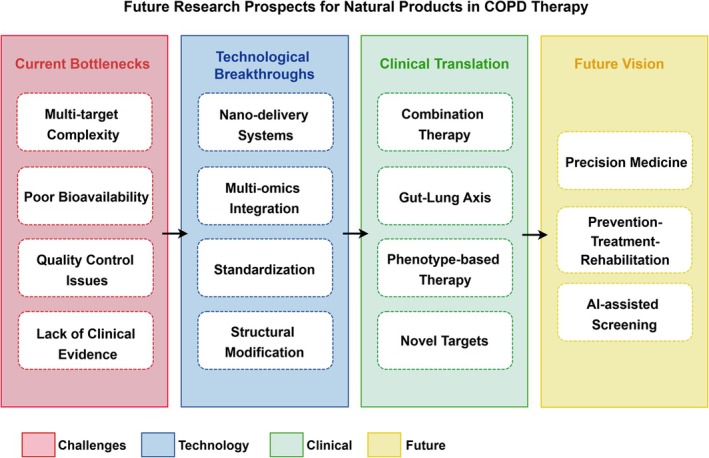
Future research prospects and potential advancements of natural products in COPD therapy (created in https://www.figdraw.com/).

To overcome these limitations and advance clinical translation, future research should focus on developing nano‐delivery systems and structural modifications to improve bioavailability, utilizing multi‐omics approaches to dissect multi‐target mechanisms, and establishing standardized quality control systems. Clinically, combination therapies, gut‐lung axis interventions, and phenotype‐based precision medicine should be explored (Figure 12). Ultimately, this will facilitate the integration of NPs into full‐cycle management strategies encompassing prevention, treatment, and rehabilitation of COPD.

### 7.5 Limitations of Current Evidence

First, the majority of evidence comes from preclinical studies using animal models (CS/LPS/CSE/PM_2_._5_/PPE‐induced COPD models), which may not fully recapitulate the complex pathophysiology and comorbidities of human COPD. Second, significant heterogeneity exists in study designs, dosing regimens, duration of treatment, and outcome measurements across different studies, making direct comparisons challenging. Third, many NPs suffer from poor bioavailability and lack standardized extraction protocols, leading to batch‐to‐batch variations in chemical composition and biological activity. Fourth, clinical trials are limited in number and sample size, with most focusing on short‐term outcomes rather than long‐term disease progression or mortality. Finally, potential publication bias favoring positive results may exist in the NP research field. Future studies should address these limitations through well‐designed randomized controlled trials with standardized formulations and clinically relevant endpoints.

## Author Contributions

**Xiaoju Liu:** writing – review and editing. **Shuna Wei:** writing – original draft.

## Funding

This work was supported by the National Natural Science Foundation of China (82460009) and the Gansu Province Key Research and Development Program (22YF7FA083).

## Ethics Statement

The authors have nothing to report.

## Consent

The authors have nothing to report.

## Conflicts of Interest

The authors declare no conflicts of interest.

## Data Availability

The authors have nothing to report.
